# The functional TNF-α^−308^G > a single-nucleotide polymorphism (rs1800629): association with the predictive indices of breast cancer carcinogenesis

**DOI:** 10.1007/s10549-024-07536-y

**Published:** 2024-11-21

**Authors:** Sherif Refaat, Hanan E. Al-Rashidi, Rania A. Abd El Azeem, Walaa E. Nouh, Sahar Hamed, Zeinab R. Attia

**Affiliations:** 1 Department of Medical Oncology, Oncology Center, Mansoura University, Mansoura, Egypt; 2 Medical Laboratory Technology Department, College of Applied Medical Science, Taibah University, Madinah, Saudi Arabia; 3 Mansoura University Children’s Hospital, Mansoura University, Mansoura, Egypt; 4 Urology and Nephrology Center, Mansoura University, Mansoura, Egypt; 5 Department of Clinical Laboratory Sciences, College of Medical Applied Sciences, University of Hafr Al Batin, Hafr Al Batin, Saudi Arabia

**Keywords:** Breast cancer, TNF-α, Single-nucleotide polymorphism, Carcinogenesis, Risk factor, Predictive index

## Abstract

**Background:**

Compared with all other cancer types, Breast cancer (BC) among women has now exceeded them all as the primary reason for cancer worldwide. The BC represents 11.7% of all cancer cases and accounts for a predestined 2.3 million new cases. It is the fourth primary reason for cancer-associated deaths in women. With a staggering 200–400% increase in the relative incidence of BC in Egypt, there is an urgent need for new diagnostic or predictive markers.

**Purpose:**

The current investigation aims to explore the connection of the functional TNF-α^−308^G > A (rs1800629) single-nucleotide polymorphism (SNP) with different breast cancer predictive indices.

**Methods:**

The ARMS-PCR method was used for genotyping TNF-α^−308^G > A SNP. Three groups were recruited for the study: 79 patients with benign breast inflammation (BBI); 163 with breast cancer (BC) and 144 controls (C).

**Results:**

The TNF-α^−308^G > A SNP was distributed among different groups in a unique pattern; in the control group 63.9% of cases were in the GG, 34% were in the GA, and 2.1% were in the AA. The BC group had 14% GG, 79% GA, and 7% AA, while the BBI group had 24% GG, 76% GA, and 0% AA. The AA genotype and A allele represented a strong significant correlation with risk factors in the BC group (OR_AA_: 14.67 [95% CI = 3.78–56.91] and OR_A_: 0.27 [95% CI = 0.19–0.39], respectively; *P* < 0.0001) in contrast to the control group. However, in the BBI group, a strong significant correlation was noted with the GA genotype (OR_GA_: 5.93 [95% CI = 3.18–11.04] *P* < 0.0001). In the BC group, the AA genotype shows a significant increase in Nottingham Prognostic Index (NPI) in positive ER and PR in contrast to the relevant negative ones (*P* = 0.02 and 0.002, respectively). However, the GA genotype significantly increased NPI in positive Her2 and metastatic patients (*P* = 0.03 and 0.01, respectively).

**Conclusion:**

This research is the first to correlate TNF-α^−308^G > A (rs1800629) SNP in Egyptian BC patients. The A allele, GA & AA genotypes, and the Overdominant model of the TNF-α^−308^G > A gene variants were recorded as prognostic risk factors for BC carcinogenesis.

**Supplementary Information:**

The online version contains supplementary material available at 10.1007/s10549-024-07536-y.

## Introduction

Breast cancer (BC) has the greatest incidence among Egyptian female cancer types; National Cancer Institute (NCI) in Egypt, reported more than 32% of total cancer patients were diagnosed with BC, and a triple rise in BC incidence is anticipated to occur by 2050 [1]. Egyptian BC patients have an increased death rate than Americans and the West. Approximately 85% of all diagnosed BCs had no family history. For Egyptian women, BC is the second most typical reason for mortality from cancer. This could be explained by the genetic changes brought on by aging or lifestyle choices, which typically manifest at later stages in younger age groups [2, 3]. With a wide range of individual patient outcomes, BC is the most prevalent type of cancer in women worldwide and the main reason for cancer-related deaths. In 2020, BC saw 685,000 deaths and over 2.3 million new cases. According to estimates by 2040, the number of new cases and annual fatalities from BC would surpass 3 million, primarily as a result of delayed diagnosis and limited access to appropriate treatment. Global measures are required to combat its increasing burden, in developing countries where incidence is rapidly increasing and fatality rates are great [4–8].

Accurately forecasting the course of breast cancer is crucial for researchers, doctors, and patients alike. Numerous prognostic models have been created for BC, but only a small amount has been widely validated in various contexts. Crucially, these devices’ efficacy are subpar in independent populations, especially in high-risk patients and older and younger patients [9, 10]. As a result of developments in genomic analysis over the past ten years, BC has been split into intrinsic molecular subtypes, with the St. Gallen Consensus dividing it into various molecular subtypes. These subtypes are luminal A-like [estrogen receptor (ER)^+ve^, progesterone receptor (PR)^+ve^, human epidermal growth factor receptor 2 (Her2)^−ve^ Ki-67 low] (ER^+ve^PR^+ve^Her2^−ve^); luminal B-like model (ER^**+ve**^**,** PR^**+ve**^, Her2^**+ve**^), Her2-enriched model (ER^−ve^PR^−ve^Her2^+ve^), and triple-negative BC (TNBC) (ER^−ve^PR^−ve^Her2^−ve^), [11, 12].

Accurate guidelines, calculators, and prognostic and predictive indices are necessary for personalized medicine in BC. Numerous such tools have been created to assess relapse risk and make adjuvant therapy decisions for patients with early-stage BC. In addition to genetically profiling malignant tumors, this may result in the creation of targeted treatments that can pave the way for efficient treatment plans. This goal can help to avoid patient overtreatment [13, 14]. Patient age, the status of menopause, histological tumor size, lymph node status, tumor grade, and expression parameters for ER, PR, Her2, and Ki-67 by immunohistochemistry (IHC) were the most common prognostic factors for BC relapses. These parameters are essential for developing precise treatment plans and assessing prognosis and outcome. The Nottingham Prognostic Index (NPI), which was established in 1978, is one of these risk categories. This scale computes a score based on histology grade, lymph node stage, and tumor size; the five-year survival rate is then ascertained using this score [15, 16]. The luminal A-like group was predicted to have a favorable prognosis, but the TNBC and Her2-enriched groups were predicted to have an unfavorable prognosis. In comparison to the luminal A-like group, a less favorable prognosis was anticipated for the luminal B-like group. A comprehensive prognostic model has been reviewed [17]. The issue in using molecular predictive genes lies in determining its functional measure where the differences between different models can be easily detected and individualized treatment goals can be achieved [18, 19].

The identification and elimination of cancer are significantly aided by the immune system. In the immune system’s reaction to cancer cells, cytokines are essential players. Immune and stromal cells produce cytokines, which are low molecular weight proteins that facilitate cell-to-cell communication. They are also engaged in the control of cell migration, apoptosis, proliferation, survival, and differentiation. Activation status of surrounding cells, pro- and anti-inflammatory cytokine balance, receptor expression content, and chronic inflammation have all been linked to the induction of malignancy and cell transformation by these proteins. Cytokines also affect the antitumor response. These cytokines stimulate several signaling pathways, such as those involved in metastasis, metabolism, angiogenesis, proliferation, and apoptosis to induce tumors [20–23]. It may be possible to describe BC and the immunological makeup of the microenvironment in it by monitoring circulating cytokine levels. Elevated levels of Th2-related and proinflammatory cytokines in serum may be a sign of aggressive and more advanced-stage BC and may be linked to a poorer prognosis. Circulating cytokines can thus be employed as intriguing non-invasive prognostic indicators [20, 24–26]. It was demonstrated that the cytokine level profile in the tumor was connected to functional SNPs in the cytokine gene. Cytokines and receptors SNPs can frequently occur in noncoding areas like promoters and introns or in coding sections, which are less common [27–29].

Proinflammatory cytokine TNF-α has been linked to several biological processes, comprising survival, differentiation, and cell proliferation. Although many different types of cells can create it, macrophages are the primary producers of TNF-α. T and B lymphocytes, fibroblasts, tumor cells, keratinocytes, neutrophils, endothelial cells, neurons, mast cells, and NK cells are also TNF-α producers. It has been observed that TNF-α facilitates carcinogenesis through chronic inflammation [20, 30]**.** The TNF-α gene can be found in the major histocompatibility complex class III region at chromosomal region 6p21.3. A type II transmembrane protein of 233 amino acids that produces stable homotrimers is encoded by its gene. The TNF-α gene’s promoter region contains the most investigated SNPs (^−1031^ T/C, ^−863^C/A, ^−857^C/T, ^−308^G/A, and ^−238^G/A). It has been reported that these SNPs regulate TNF-α production. High TNF-α levels have been linked to the TNF-α promoter alleles TNF-α^−308^A and TNF-α^−238^A. TNF-α^−308^G > A SNPs affect the overall survival of cancer patients and represent a possible target for cancer therapies [30–33]. The sample size restrictions and the absence of a correlation between the genotype and clinical cancer prognosis have been noted as limitations.

Studies on genetic epidemiology have shown a connection between cytokine polymorphisms and cancer prognosis. The TNF-α gene’s promoter region (− 308) has a G-to-A substitution (TNF-α^−308^G > A), which increases TNF-α expression and is linked to several illnesses, including hepatocellular and other cancers [34–36]. The TNF-α^−308^G > A SNP's relationship to cancer risk or prognosis has been the subject of several research, but it is still debatable if this SNP is a useful predictor of cancer patients’ overall metastasis and survival. The correlation between particular immune cell types in the tumor microenvironment and serum cytokine levels in BC is a crucial consideration when assessing these levels. TNF-α cytokines are present at higher levels in healthy A allele carriers than in G allele carriers. It has been established that the G-to-A substitution polymorphism directly affects the generation of TNF-α [37, 38].

The correlation between these polymorphisms and BC has been reported in multiple studies, showing the impact of different cytokine gene polymorphisms [39–43]. However, the associations of TNF-α gene SNPs with BC susceptibility, prognosis, and tumor characteristics are still debated. These findings indicate that TNF-α^−308^G > A (rs1800629) SNP may be linked to risk factors for BC development in the USA, Morocco, and Iraqi populations [33, 44, 45]. The contentious function of these SNPs in BC has continued. This work aimed to determine the association of functional TNF-α^−308^G > A (rs1800629) SNP to BC characteristics and its ability to predict BC carcinogenesis.

## Materials and methods

### Ethical declaration

One hundred and sixty-three newly diagnosed female breast cancer patients and seventy-nine patients with benign breast inflammation were evaluated at Mansoura University Oncology Centre in 2019 and 2020. The diagnosis was made using fine needle aspiration cytology, mammography, and histopathology. Exclusion criteria are restricted to any other cancer types or patients with major cardiac, renal, hepatic, skeletal, or neurological disorders. Patients with infections were also excluded from the study. The Mansoura University Institutional Review Board (IRB) authorized the protocol under the number (R.22.031651R1.R2), The guidelines and regulations of WMA and the Helsinki Declaration technique were used [46–48]. Every participant gave their informed consent. All patient-related information, including biological samples, was anonymized to maintain privacy.

### Patients and controls

The age range of the 163 female BC patients was 27 to 80 years old, with a median age of 52.7 years. Tumor size, PR, ER, and Her2 status were determined from the routine hospital patient’s datasheet for each patient. The TNF-α-308G > A (rs1800629) SNP in the BC group was further linked with the specific prognostic variables in each individual. The BC patients were recently found to have breast cancer without the need for chemotherapy or radiation treatment. The NPI, a compulsory prognostic score that reliably forecasts survival for individuals with BC [49, 50]**,** was determined for every patient. The cut-off points were used to divide the three prognostic groups. A good prognosis index (GPI) is defined as an NPI < 3.4, a moderate prognostic index (MPI) as an NPI of 3.41–5.4, and a poor prognostic index (PPI) as an NPI > 5.41. The equation used for NPI quantitation is.

$${\text{NPI}} = \left( 0.2\, \times \,{\text{tumor size}} \right)\, + \,{\text{Node status}}\, + \,{\text{Grade status}}.$$

Two more groups were added to the roster. The median age of 45.9 years (range: 36–63 years) was provided in the control group (C), 144 volunteers recruited from Mansoura University as solid organ donors and 79 patients with benign breast inflamed cells (BBIs) and cancer-free, respectively.

### DNA extraction and TNF-α^−308^G > A (rs1800629) genotyping

The blood samples were collected in EDTA-contained tubes. After spinning at 2500×*g* for 9 min at room temperature, puffy coats of EDTA samples were recovered, and DNA was extracted from them. Promega DNA Extraction Kit (Promega. USA. A1120) instructions were followed to extract the DNA. Using a Nanodrop spectrophotometer, the absorbance ratio 260/280 was used to determine the DNA’s purity and amount.

Using the amplification refractory mutation system-polymerase chain reaction (ARMS-PCR) approach, the genetic variant of the TNF-α^−308^G > A (rs1800629) genotype was identified, as per Ahmed et al. [51]**.** Utilizing the Thermo Scientific ARKTIK thermal cycler, genomic DNA was amplified for polymorphism analysis. Three distinct primers were used in the ARMS-PCR for the TNF-α^−308^G > A SNP: one forward primer for the G allele, one forward primer for the A allele, and one common reverse primer. These primers were all made by Eurofins (Genomics, Germany). For the TNF-α^−308^G > A (rs1800629) genotyping, two distinct PCR mix tubes containing 20 μL of each allele were used. Approximately 200 ng of genomic DNA, 2 μL of each primer (10 pmol/ml), 2 μL of nuclease-free water, and 10 μL of 2X ViRed Taq Master Mix (Vivantis, Malaysia) were used in the mixture.

All the participants, including patients and healthy controls, were tested two times. One PCR was carried out employing a common reverse primer (5′-TCTCGGTTTCTTCTCCATCG-3′), and the forward primer was used for the G genotype (5′-ATAGGTTTTGAGGGGCATGG-3), the second PCR was carried out by the identical common primer and the forward primer that represented the A genotype (5′-ATAGGTTTTGAGGGGCATGA-3′). The following were the thermal conditions: 5 min at 94 °C; 10 cycles of annealing for 50 s at 65 °C, denaturing for 15 s at 94 °C, and extending for 40 s at 72 °C. Next, 25 cycles of 20 s at 94 °C for denaturation, 50 s for annealing at 59 °C, and 50 s for extension at 72 °C were employed. Seven minutes more were applied for the last extension at 72 °C.

**Fig. 1:**
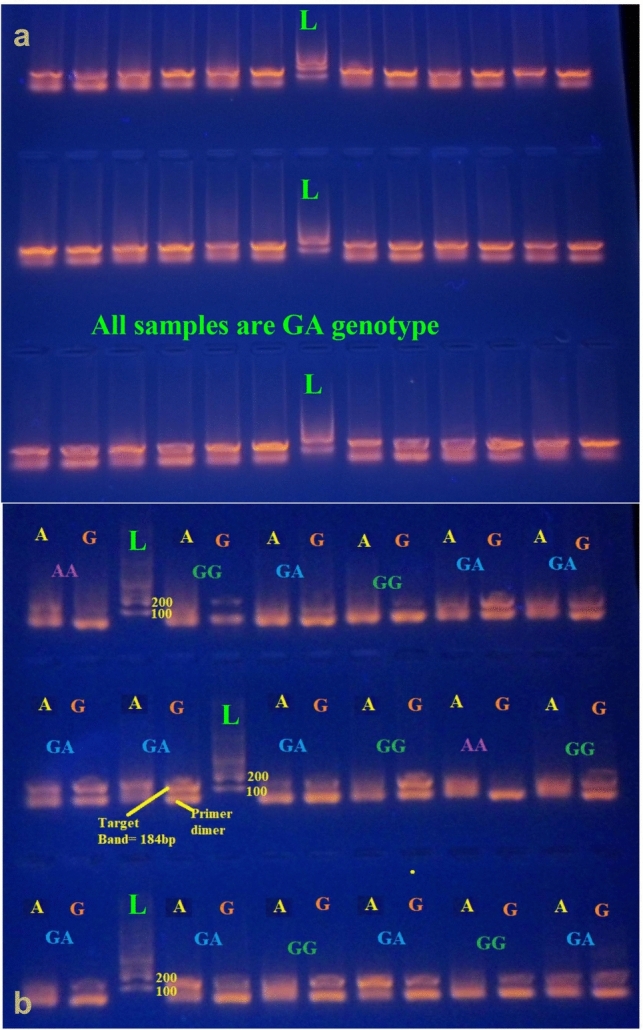
Agarose gel electrophoresis of the TNF-α^−308^G > A (rs1800629) different genotypes. The band of each A or G allele was detected at 184 bp. Lane L indicates DNA Ladder 100 bp marker (Vivantis, Malaysia), each genotype represented with two lanes, one for the A allele and the other one for the G allele. **a** represents all samples with the GA genotype indicating its high percentage presence in the BC group. **b** The AA genotype is indicated when the A allele band is present alone. The GG genotype is indicated when the G allele band is present alone and the GA genotype is indicated when both allele bands are present

Electrophoresis on a 1.5% agarose gel, stained with ViSafe Red Gel Stain (Vivantis, Malaysia) was used to visualize the amplified products at 184 bp band, and imaged, from the different group samples. Forward primer G yielded the right size band, indicating the homozygote genotype GG; conversely, forward primer A, indicating homozygote genotype AA, yielded the same size band. The genotype was heterozygote GA based on the presence of the correct band from the first run with G and the second run with A for the same individuals Fig. 1**.** To verify test repeatability and uniformity, 15% of the DNA samples were chosen at random for a second PCR to ensure that there was no misinterpretation during genotyping. The outcomes were in perfect agreement with the earlier ones.

### Statistics

The power of the study was calculated using G*Power software for two independent groups with [Effect size *d* = 0.5, α err prob = 0.05, Power (1-β err prob) = 0.95, and Allocation ratio N2/N1 = 1]. The calculated minimum sample size equals 105 participants. SPSS version 16 statistical software was used to examine the data. Allelic frequencies in each of the study subjects were determined using the gene counting method. Using the chi-square test, the frequency of TNF-α-308G > A (rs1800629) genotype and allele frequency was compared between different studied groups. The 95% confidence intervals (CIs) and odds ratios (ORs) were used to determine the relative risk for BC. Utilizing the same methods, the correlation values between the TNF-α^−308^G > A (rs1800629) genotype in BC patients and the histological and clinical data were determined. Using a two-tailed Student’s t-test, the NPI was quantitatively compared to that of the TNF-α^−308^G > A (rs1800629) genotype. It was considered that statistical significance was demonstrated by *P* < 0.05.

## Results

### Distribution of the TNF-α^−308^G > A (rs1800629) genotypes in different studied groups

**Fig. 2:**
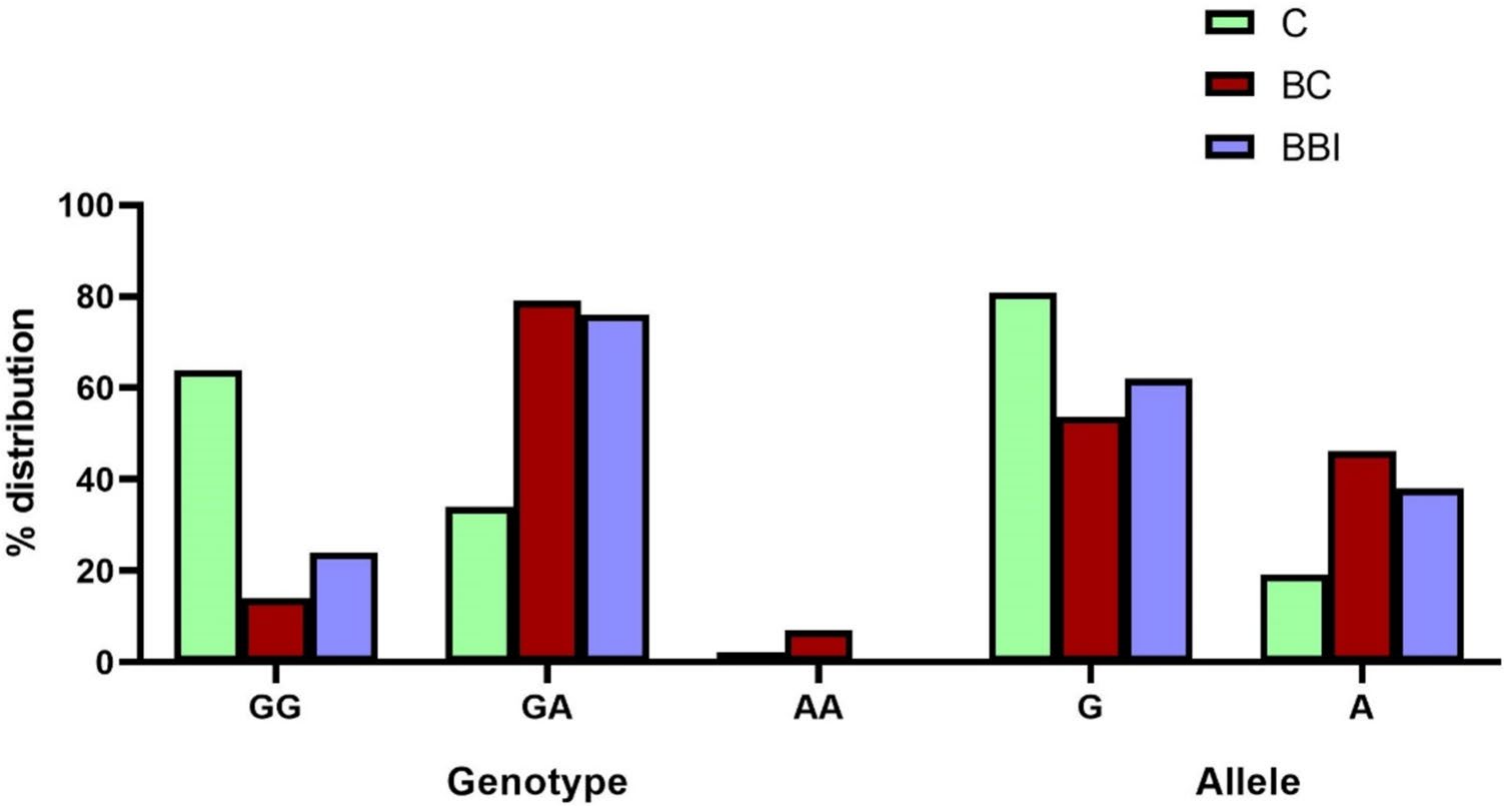
Distribution percent of the TNF-α^−308^G > A (rs1800629) genotypes and alleles in different study groups

The study comprised 260 female patients with breast involvement, of which 163 belonged to the breast cancer (BC) group and 97 to the benign breast inflamed cells (BBI) group. Additionally, 144 unrelated healthy controls (C) from the same region were included in the study. As shown in Fig. 1, the band of amplified PCR product for TNF-α-308G > A (rs1800629) SNP is presented at 184 bp. Based on these findings, TNF-α-308G > A SNP genotypes and alleles in various groups under investigation were identified and assessed to the corresponding healthy controls. The outcomes are displayed in Table 1 and Fig. 2. Different TNF-α genotypes and different TNF-α^−308^G > A genetic models were calculated for the studied groups. In the dominant TNF-α^−308^G > A model the GG genotype versus those with (AA + GA genotypes), there was a strong significant risk factor for A allele in both the BC and BBI groups (*P* < 0.0001) in terms of quantity of cases in total in contrast to their respective controls and a significant risk factor to A allele in the BC in comparison to the BBI groups (*P* = 0.043), [ORs (95% CI) were 10.77 (6.17–18.79), 5.59 (3.01–10.36), and 0.52 (0.26–1.02), respectively].

**Table 1:** Distribution of the TNF-α^−308^G > A (rs1800629) genotype frequencies (genetic models) with risk estimate in different study groups

TNF Genotype	Group's # (%)					
	Control 144		BC 163		BBI 79	
GG (REF)	92 (63.9)		23 (14)		19 (24)	
GA	49 (34)		129 (79)		60 (76)	
AA	3 (2.1)		11 (7)		0 (0)	
Allele						
G	233 (80.9)		175 (53.7)		98 (62)	
A	55 (19.1)		151 (46.3)		60 (38)	
Statistics	GG vs AA + GA (dominant)					
		C vs BC		C vs BBI		BC vs BBI
OR		10.77		5.59		0.52
95% CI		(6.17–18.79)		(3.01–10.36)		(0.26–1.02)
P		0.0001		0.0001		0.043
	AA vs GG + GA (recessive)					
OR		0.29		1.56		1.52
95% CI		(0.08–1.07)		(1.41–1.72)		(1.38–1.67)
P		0.044		0.26		0.012
	GA vs AA + GG (overdominant)					
OR		0.13		0.16		1.2
95% CI		(0.08–0.23)		(0.09–0.31)		(0.63–2.72)
P		0.0001		0.0001		0.34
	A allele vs G allele					
OR		0.27		0.38		1.41
95% CI		(0.19–0.39)		(0.25–0.59)		(0.95–2.08)
P		0.0001		0.0001		0.050
	GG vs GA (codominant)					
OR		10.53		5.93		0.56
95% CI		(5.99–18.49)		(3.18–11.04)		(0.28–1.11)
P		0.0001		0.0001		0.07
	GG vs AA (homozygote)					
OR		14.67		0.83		0.55
95% CI		(3.78–56.91)		(0.76–0.9)		(0.42–0.72)
P		0.0001		0.57		0.004

GG = wild-type homozygote genotype (dominant),

AA = mutant homozygote genotype (recessive),

GA = heterozygote genotype

*OR* Odds Ratio, *CI* Confidence Interval, *REF* Reference Genotype,

P = Significance

The recessive model of TNF-α^−308^G > A (AA genotype versus GG + GA genotype) was less significantly connected to the BC group (*P* = 0.044) and with no significance to the BBI group (*P* = 0.26) for the total number of cases in contrast to their respective controls. Moreover, there was a risk factor to AA genotype in the BC when compared to BBI groups (*P* = 0.012), [ORs (95% CI) were 0.29 (0.08–1.07), 1.56 (1.41–1.72), and 1.52 (1.38–1.67), respectively]. The allelic frequency of G vs A was significant with a high risk of A allele in the BC group when compared to the C group (*P *< 0.0001) and to the BBI group (*P* = 0.050), [ORs (95% CI) were 0.27 (0.19–0.39), 0.38 (0.25–0.59), and 1.41 (0.95–2.08), respectively].

Examining the codominant model (GG vs. GA) showed that there was a high-risk association with the GA genotype (*P* < 0.0001) for both the BC and BBI groups in contrast to the healthy control group. However, the homozygote model (GG vs. AA) showed that there was a risk factor for the AA genotype in the BC group when compared to the BBI and C group (*P* < 0.004, *P* < 0.0001, respectively). All the information regarding the TNF-α-308G > A (rs1800629) SNP for each of the participants under study is collected in Table 1**.** The findings provide credence to the possibility that the TNF-α-308G > A (rs1800629) SNP contributes to the development of breast cancer in the Egyptian population under investigation.

### Distribution of the TNF-α^−308^G > A (rs1800629) genotype among different tumor variants in the BC group

The study participants’ demographic, clinicopathological, and biomarker information were gathered from the patient's medical records and are displayed in Table 2, First Column. The table displays various features that correspond to the number and percentage of each variant for the 163 patients in the BC group. Of these features, stage II was the most common cancer stage (67.5%), node status was N0 (34.4%), cancer grade was grade II (71.2%), tumor size was ≥ 2–5 cm (74.2%), NPI was > 3.4–5.4 (MPI, 74.2%), positive ER was (79.8%), positive PR was (76.1%), negative Her2/neu expression was (54.6%), negative metastasis was (85.3%) and left operated breast was (61.4%), and age over 50 years was (55.2%). Out of the various TNF-α-308G > A (rs1800629) genotypes in the BC group, GG, GA, and AA were present in (14.1%, 79.1%, and 6.7%), respectively.

**Table 2:** Distribution of the TNF-α^−308^G > A (rs1800629) genotype frequencies in different tumor variants in the BC group (163 patients)

Variables	Patient number (percentage)		
Genotype	GG	GA	AA
	23 (14.2)	129 (79.1)	11 (6.7)
Cancer stage			
26 (15.9) T1	2 (8.7)	24 (18.6)	0 (0)
110 (67.5) T2	19 (82.6)	84 (65.1)	7 (63.6)
21 (12.9) T3	1 (4.3)	16 (12.4)	4 (36.4)
6 (3.7) T4	1 (4.3)	5 (3.9)	0 (0)
Node status			
56 (34.4) N0	8 (34.8)	46 (35.7)	2 (18.1)
42 (25.8) N1	5 (21.7)	34 (26.4)	3 (27.3)
40 (24.5) N2	8 (34.8)	27 (20.8)	5 (45.5)
25 (15.3) N3	2 (8.7)	22 (17.1)	1 (9.1)
Overall grade			
3 (1.8) G1	0 (0)	1 (0.8)	2 (18.2)
116 (71.2) G2	16 (69.6)	93 (72.1)	7 (63.6)
44 (27) G3	7 (30.4)	35 (27.1)	2 (18.2)
Tumor Size			
14 (8.6) < 2 cm	1 (4.3)	13 (10.1)	0 (0)
121 (74.2) 2- 5 cm	19 (82.6)	94 (72.9)	8 (72.7)
28 (17.2) > 5 cm	3 (13.1)	22 (17)	3 (27.3)
Nottingham prognostic index (NPI)			
9 (5.5) > 2.4- 3.4	2 (8.7)	6 (4.7)	1 (9.1)
121 (74.2) > 3.4- 5.4	14 (60.9)	98 (76)	9 (81.8)
33 (20.3) > 5.4	7 (30.4)	25 (19.3)	1 (9.1)
Estrogen receptor (ER)			
33 (20.2) Negative	2 (8.7)	27 (20.9)	4 (36.4)
130 (79.8) Positive	21 (91.3)	102 (79.1)	7 (63.6)
Progesterone receptor (PR)			
39 (23.9) Negative	3 (13)	34 (26.4)	2 (18.2)
124 (76.1) Positive	20 (87)	95 (79.6)	9 (81.8)
Her2/neu expression			
89 (54.6) Negative	13 (56.5)	69 (53.5)	7 (63.6)
74 (45.4) Positive	10 (43.4)	60 (46.5)	4 (36.4)
Metastasis			
139 (85.3) Negative	22 (95.7)	108 (83.7)	9 (81.8)
24 (14.7) Positive	1 (4.3)	21 (16.3)	2 (18.2)
Operation site			
100 (61.4) Lt MRM	15 (65.2)	78 (60.5)	7 (63.6)
63 (38.6) Rt MRM	8 (34.8)	51 (39.5)	4 (36.4)
Age (year)			
73 (44.8) < 50y	12 (52.2)	56 (43.4)	5 (45.5)
90 (55.2) > 50y	11 (47.8)	73 (56.6)	6 (54.5)

The distributions of different genotypes of TNF-α^−308^G > A (rs1800629) gene among different tumor variables in BC patients (163 patients) are described in depth in Table 2. Among all the most common different tumor variables, the GA genotype was found to be the most prevalent genotype. The percentage of patients with the GG genotype in the BC group (14.2%) was greater than that with the AA genotype (6.7%). However, the AA genotype is always predominant in the worse variables of T3, N2, > 5 cm tumor size, > 3.4–5.4 NPI, negative ER and PR, positive Her2new, and metastasis. The GG genotype tended to be predominant in the worse variables of G3 and > 5.4 NPI.

The detailed distributions of different genotypes of TNF-α^−308^G > A (rs1800629) gene in response to different tumor variables in BC patients (163 patients) are illustrated in Supplemental Tables 1–9. As an example, for this distribution, the NPI was selected to represent different TNF-α^−308^G > A genotypes associated with the different NPI types within all the variables studied (Table 3). The GA genotype was most common in the BC group (79%). For the GA genotype, the most common variables were MPI (75.9%) followed by PPI (19.4%) and GPI (4.6%). In response to the cancer stage, T2 was ~ 68% for both MPI and PPI. In response to node status, N0 was 40% in the MPI, and N3 was 40% in the PPI. In response to cancer grade, G2 had an 87% in MPI, and G3 had an 88% in PPI. In terms of tumor size, 77% of the MPIs and 64% of the PPIs were 2–5 cm long. In response to Estrogen receptors, 80% of the MPIs and 76% of the PPIs were positively expressed. In response to the progesterone receptor, 76% of the MPIs and 60% of the PPIs were positively expressed. In response to Her2/neu protein expression, 57% of the MPIs were negative, while 60% of the PPIs were positive. In response to Metastasis, 13% of patients were positive for MPI and 32% were positive for PPI. In response to patients’ ages, ~ 56% of the patients were aged older than 50 years in both the MPI and PPI groups.

**Table 3:** Distribution of the TNF-α^−308^G > A (rs1800629) genotype frequencies in different tumor variants in the BC group (163 Patients) in response to the Nottingham Prognostic Index (NPI)

Variables	GG^GP^	GG^MP^	GG^PP^	GA^GP^	GA^MP^	GA^PP^	AA^GP^	AA^MP^	AA^PP^
NPI	2	14	7	6	98	25	1	9	1
Cancer stage									
26 (15.9) T1	2	0	0	6	18	0	0	0	0
110 (67.5) T2	0	13	6	0	67	17	1	6	0
21 (12.9) T3	0	0	1	0	12	4	0	3	1
6 (3.7) T4	0	1	0	0	1	4	0	0	0
Node status									
56 (34.4) N0	2	6	0	6	40	0	1	1	0
42 (25.8) N1	0	3	2	0	28	6	0	2	1
40 (24.5) N2	0	5	3	0	18	9	0	5	0
25 (15.3) N3	0	0	2	0	12	10	0	1	0
Overall grade									
3 (1.8) G1	0	0	0	1	0	0	1	1	0
116 (71.2) G2	2	14	0	5	85	3	0	7	0
44 (27) G3	0	0	7	0	13	22	0	1	1
Tumor Size									
14 (8.6) < 2 cm	1	0	0	4	8	1	0	0	0
121 (74.2) 2- 5 cm	1	12	6	2	76	16	1	7	0
28 (17.2) > 5 cm	0	2	1	0	14	8	0	2	1
Estrogen receptor (ER)									
33 (20.2) Negative	0	2	0	2	19	6	1	3	0
130 (79.8) Positive	2	12	7	4	79	19	0	6	1
Progesterone receptor (PR)									
39 (23.9) Negative	0	3	0		24	10	1	1	0
124 (76.1) Positive	2	11	7	6	74	15	0	8	1
Her2/neu expression									
89 (54.6) Negative	1	9	3	3	56	10	1	6	0
74 (45.4) Positive	1	5	4	3	42	15	0	3	1
Metastasis									
139 (85.3) Negative	2	14	6	6	85	17	1	7	1
24 (14.7) Positive	0	0	1	0	13	8	0	2	0
Operation site									
100 (61.4) Lt MRM	1	10	4	3	61	14	0	6	1
63 (38.6) Rt MRM	1	4	3	3	37	11	1	3	0
Age (year)									
73 (44.8) < 50y	1	9	2	3	42	11	0	5	0
90 (55.2) > 50y	1	5	5	3	56	14	1	4	1

GPI =  > 2.4–3.4; MPI =  > 3.4–5.4; PPI =  > 5.4

### Risk estimates for the TNF-α^−308^G > A (rs1800629) genotype model for different variants and different models of the BC group

By comparing the different TNF-α^−308^G > A (rs1800629) genotype models as risk estimates with different tumor characteristics in the BC group, the results revealed that the TNF-α^−308^G > A (rs1800629) genotype had no connection with any of the other tumor variants (Supplemental Tables 10- 12). Table 4 shows that the risk estimated was not affected by the different TNF-α^−308^G > A (rs1800629) genotype models in response to the NPI in the BC group.

**Table 4:** Distribution of the TNF-α^−308^G > A (rs1800629) genotype frequencies (genetic models) with risk estimate in response to NPI in the BC group

Model	Genotype # (%)			OR (95% CI)	P
Codominant	MPI 121 (78.6)	PPI 33 (21.4)			
GG	14 (11.5)	7 (21.2)		1	
GA	98 (80.9)	25 (75.7)		0.51 (0.18–1.39)	0.14
AA	9 (7.4)	1 (3.1)		0.22 (0.02–2.12)	0.17
Dominant	AA + GA vs GG			0.48 (0.17–1.32)	0.12
Recessive	AA vs GG + GA			2.57 (0.31–21.06)	0.32
Overdominant	GA vs AA + GG			1.36 (0.54–3.41)	0.32
Allele G Allele A	117 (48.1)		39 (59.1)	0.64 (0.37–1.12)	0.075
	126 (51.9)		27 (40.9)		

When testing the host TNF-α^−308^G > A (rs1800629) genotype model in different BC prognostic models [11, 12], the poor prognostic model of the **luminal B model, (ER**^**+ve**^**PR**^**+ve**^**Her2**^**+ve**^**)** (52 patients), the very poor prognostic model (triple^–ve^ model), which shows the negative expression for different hormonal status and Her2 protein (11 patients), and the very poor prognostic hormonal status **Her2-enriched model (ER**^**−ve**^**PR**^**−ve**^**Her2**^**+ve**^, 14 patients), showed no statistically significant risk estimate within different host TNF-α^−308^G > A (rs1800629) genotype models when in contrast to the good prognostic model (64 patients) **luminal A model (ER**^**+ve**^**PR**^**+ve**^**Her2**^**−ve**^, Supplemental Table 14). When testing the triple^−ve^ model vs the Her2-enriched model, no risk estimate was noted (Supplementary Table 13).

### Distribution of the TNF-α^−308^G > A (rs1800629) genotype according to the prognostic NPI in the BC group

For the 163 BC patients for whom adequate clinical data were available, the NPI was computed. 9 patients were in the GPI group, 121 patients were in the MPI group, and 33 patients were in the PPI group, their NPI ranged from 2.4 to 6.4. Patients. The frequency of different TNF-α^−308^G > A (rs1800629) genotypes according to the means and standard errors of the means calculated for the NPIs are presented in Table 5. A significant increase was noticed within different genotypes when utilizing the student’s t-test for the MPI (P = 0.011).

**Table 5:** Means and standard error of the means of NPI for the TNF-α^−308^G > A (rs1800629) genotype of the BC group and within different prognostic groups

	TNF	N	Mean	SEM	Sig		TNF	N	Mean	SEM	Sig
NPI	GG	23	4.70	0.21		MPI	GG	14	4.29	0.17	
	GA	129	4.67	0.07	0.895		GA	98	4.46	0.05	0.234
	AA	11	4.85	0.25	0.642		AA	9	4.92	0.14	0.011
	P1 0.469						P1 0.013				
GPI	GG	2	3.40	0.01		PPI	GG	7	5.88	0.07	
	GA	6	3.17	0.15	0.205		GA	25	5.85	0.05	0.712
	AA	1	2.90				AA	1	6.20	0.17	0.183
	P1 0.547						P1 0.185				

*Sig.* significance of GG genotype vs other two genotypes, *P1* significance of GA genotype vs AA genotype, *GPI* good prognosis index is defined as has NPI < 3.4, *MPI* = moderate prognostic index as has NPI of 3.41–5.4, *PPI* poor prognostic index as has NPI > 5.41

The detailed significance of the calculated NPIs is summed up in Table 6 for hormonal status, Her2 protein, and metastasis in patients with different TNF-α^−308^G > A (rs1800629) genotypes. A significant rise in calculated NPI was noted in the AA genotype in ER^+ve^ and PR^+ve^ when in contrast to the negative ones (P = 0.02 and 0.002, respectively), while in the GA genotype, a significant decrease was noted in PR^+ve^ when compared to the negative one (P = 0.039). However, regarding Her2 protein expression and metastasis, significant increases were noted for Her2^+ve^ and positive metastasis in the GA genotype (P = 0.035 and 0.016, respectively).

**Table 6:** Means and standard error of the mean of NPI for different hormonal markers ER, PR, Her2 protein expression, and Metastasis in the TNF-α^−308^G > A (rs1800629) genotype in BC group

Estrogen receptor					Progesterone receptor				
TNF	N	Mean	SEM	Sig	TNF		Mean	SEM	Sig
GG ER^−ve^ ER^+ve^	2	4.10	0.50		GG PR^−ve^ PR^+ve^	3	4.06	0.29	
	21	4.75	0.22	0.380		20	4.79	0.22	0.105
GA ER^−ve^ ER^+ve^	27	4.73	0.16		GA PR^−ve^ PR^+ve^	34	4.91	0.14	
	102	4.66	0.07	0.692		95	4.59	0.07	**0.039**
AA ER^−ve^ ER^+ve^	4	4.12	0.45		AA PR^−ve^ PR^+ve^	2	3.45	0.55	
	7	5.27	0.17	**0.020**		9	5.16	0.15	**0.002**
Her2/neu					Metastasis				
GG Her^−ve^ Her^+ve^	13	4.71	0.27		GG Met. ^−ve^ Met. ^+ve^	22	4.64	0.21	
	10	4.68	0.32	0.935		1	6.00		0.18
GA Her^−ve^ Her^+ve^	69	4.54	0.09		GA Met. ^−ve^ Met. ^+ve^	108	4.60	0.07	
	60	4.82	0.10	**0.036**		21	5.04	0.17	**0.016**
AA Her^−ve^ Her^+ve^	7	4.68	0.31		AA Met. ^−ve^ Met. ^+ve^	9	4.84	0.31	
	4	5.15	0.45	0.409		2	4.90	0.10	0.87

### Distribution of the TNF-α^−308^G > A (rs1800629) genotype concerning metastasis in the BC group

Metastasis, the most common complication in BC, was found in 24 patients (14.7%); one patient had the TNF-α^−308^G > A (rs1800629) GG genotype, 21 patients had the GA genotype, and 2 patients had the AA genotype. The most common metastases were bone metastasis in 8 patients; bone and LN metastasis in 5 patients; lung metastasis in 5 patients; bone and liver metastasis in 3 patients; and bone and lung metastasis in 2 patients. Another case involved metastasis to the brain, bone, and LN. A comprehensive exposition of the different TNF-α^−308^G > A (rs1800629) genotypes connected to metastasis and the calculated NPI for each individual is presented in Table 7.

**Table 7:** Distribution of different metastasis sites in the TNF-α^−308^G > A (rs1800629) genotype with NPI (values)

Site of metastasis	GG	GA	AA
Bone	0	7 (6.2, 4.6, 4.2, 4.8, 3.8, 6.2 & 5.6)	1 (4.8)
Bone & LN	0	5 (4.7, 4.6, 3.6, 5.2 & 6.2)	0
Bone & liver	0	2 (5.8 & 5.2)	1 (5)
Bone & lung	0	2 (5.6 & 4.6)	0
Bone & brain & LN	0	1 (5.8)	0
Lung	1 (6)	4 (4, 5.8, 4.5, 5)	0

Table 8 shows the different predictive models for BC patients according to the calculated NPI for each individual. According to the reference model, which includes a good prognosis; the **luminal A model, (ER**^**+ve**^**PR**^**+ve**^**Her2**^**−ve**^**)** was constructed (8/63 metastasis patients, 12.7%). The prognosis was very poor **(triple**^**–ve**^** model**, 2/11 metastatic patients, 18%). Poor prognostic negative hormonal status and **Her2-enriched model (ER**^**−ve**^**PR**^**−ve**^**Her2**^**+ve**^**)** were also constructed (1/14 metastasis patient, 7%). Other poor prognostic model of the **luminal B model, (ER**^**+ve**^**PR**^**+ve**^**Her2**^**+ve**^**)** was also detected (8/52 metastatic patients, 15.4%). The last 5 metastatic patients were classified as GA genotype, 3 of them had **(ER**^**+ve**^**PR**^**−ve**^**Her2**^**+ve**^**)** characteristics; 2 had bone and LN metastasis and one had bone and liver metastasis. The last 2 patients had **(ER**^**+ve**^**PR**^**−ve**^**Her2**^**−ve**^**)** characteristics; one had bone metastasis, and the other one had bone and lung metastasis.

**Table 8:** Distribution of different metastasis sites in the TNF-α^−308^G > A (rs1800629) genotype in response to the BC Predictive Models

Predictive models (N)	GG	GA	AA
	Site of metastasis (NPI calculated)		
luminal A model, (63) (ER^+ve^PR^+ve^Her2^−ve^)	1 lung (6)	4 bone (6.2, 4.6, 4.2 & 4.8), 1 bone & lung (5.6), and 1 lung (4)	1 bone (4.8)
(Triple^–ve^ model), (11)	0	1 bone & liver (5.8) and 1 lung (5.8)	0
Her2-enriched model (14) (ER^−ve^PR^−ve^Her2^+ve^)	0	1 lung (4.5)	0
luminal B model, (52) (ER^+ve^PR^+ve^Her2^+ve^)	0	3 bone & LN (4.7, 4.6 & 3.6), 2 bone (3.8 &6.2), 1 1 bone & LN & brain (5.8), and 1 lung (5)	1 bone & liver (5)
ER^+ve^PR^−ve^Her2^+ve^	0	2 bone & LN (5.2 & 6.2) and 1 bone & liver (5.2)	0
ER^+ve^PR^−ve^Her2^−ve^	0	1 bone (5.6) and 1 bone & lung (4.6)	0

## Discussion

TNF-α interaction has a crucial role in promoting tumor development, invasion, and metastasis [52]. The most widespread disease in women is BC which is considered a significant global public health concern [7, 8]. Tumor growth-promoting conditions have been the main description of inflammation. Throughout this illness, proinflammatory cytokines, such as TNF-α, are considered the most prevalent inflammatory cytokine in BC, which play a crucial role in its carcinogenesis. On the other hand, TNF-α can stimulate differentiation, activation, survival, or cell death in specific circumstances. Therefore, studying the variants of these cytokine polymorphisms and their implications for different breast cancer phenotypes is necessary for identifying their characteristics [52]. It is now well acknowledged that BC is the disease that is diagnosed most frequently worldwide and that it is the biggest reason for death attributed to cancer for women globally [5]. The prevalence of BC is rising in Egypt, where it continues to be a serious health issue without a cure. Of all female cancer cases, BC accounts for 33% and about 22,000 new cases are diagnosed annually. With the population growing, this trend is predicted to grow rapidly over the next few years. The National Cancer Institute (NCI) in Egypt recently released a prediction that a threefold increase would take place by 2050 [1, 3]. This research confirmed the association of the functional single-nucleotide polymorphism TNF-α^−308^G > A (rs1800629) with the disease prognosis of BC in Egyptian patients. This can be explained by the significant rise in calculated NPI in the AA genotype in positive hormonal individuals compared to the negative ones. While, regarding Her2 protein expression and metastasis, substantial increases in NPI were noted for Her2^+ve^ and positive metastasis in the GA genotype.

Numerous human disorders have been linked to the dysregulation of TNF-α production. A growing body of research implies that genetic variations in the TNF-α promoter region affect the protein’s translation and ensuing cancer [26, 34]. One of the most well-characterized polymorphisms is TNF-α-308G > A SNP; the wild-type is GG, and the A allele is linked to TNF-α overexpression and unfavorable clinical findings in cancer patients. In the current investigation, data extraction was done solely using several genotyping models. TNF-α^−308^G > A (rs1800629) SNP's wild-type genotype, GG, was more common in the control group than in the BC and BBI groups, whereas the GA genotype was more common in the BC and BBI groups than in the control group. The A allele was more common in the BC and BBI groups than in the control group. Yi F et al., [31] revealed that only the AA/GG genotype significantly predicts the survival of cancer patients; patients with the AA genotype at TNF-α^−308^G > A lived shorter lives than patients with the GG genotype, with no discernible heterogeneity. We determine the compulsory prognostic score NPI, which reliably forecasts survival for individuals with BC, we find that the AA genotype holds a high NPI score, especially in moderate prognostic index (MPI) and poor prognostic index (PPI). Following BC therapy, it was discovered that genetic variables affected the intensity and persistence of common symptoms [33].

To check for carcinogenesis in BC patients, another control group was the BBI group. TNF-α^−308^G > A (rs1800629) SNP analysis in the 79 BBI cohort showed similar results as in the healthy control group, with no variations in TNF-α^−308^G > A SNP in the recessive model (AA against GG + GA) or homozygote model (GG vs. AA). However, when contrasting the BBI group to the BC group, there was a highly significant variation in the TNF-α^−308^G > A SNP homozygote model (GG vs. AA), while the dominant (GG vs GA) and overdominant (GA vs AA + GG) models showed no differences in TNF-α^−308^G > A SNP between the two groups. Other genotypic models showed a significant variation between the two groups. There are no data with which to compare the outcomes of the current study, but these results confirm the strong involvement of TNF-α^−308^G > A (rs1800629) SNP in BC patient carcinogenesis and on a small basis, in BBI patients.

A thorough examination of TNF-α^−308^G > A (rs1800629) SNP in 163 Egyptian patients with BC, 79 BBI, and 144 healthy controls from the same region revealed a highly significant correlation with higher frequencies of the A allele in BC and BBI patients as compared to the control group. It was found that the relationship of the TNF-α^−308^G > A SNP within different groups varied based on the patient’s country of origin. The function of TNF-α^−308^G > A SNP in BC has also been investigated by three investigations. A Moroccan investigation, however, discovered that whereas BC patients had a higher incidence of the GG (low TNF-α producer) genotype, the AA (high TNF-α producer) genotype was more common than in controls (P < 0.0001) [44]. The results of the other study performed in Iraq showed that people with the AA (high TNF-α production) genotype are more prone to BC and have poorer BC prognoses [45]. The AA genotype in the current study had the lowest percentage in the control group and the BC group. While, the GA genotype is increased in the BC group, and the GG genotype is increased in the control group; when comparing the A allele, it was found to be associated with BC patients. Lastly, a Russian case–control study was unable to find any connection between BC patients and the TNF-α^−308^G > A SNP [53]. The three studies’ disparate outcomes could be explained by sampling error or variations in the patient groups’ ethnic backgrounds. In an analysis of the TNF-α^−308^G > A SNP, this SNP may be a viable target for cancer therapy as it has been shown to impact the overall survival of various cancer patients [31]. It also correlates with a lower death rate for all reasons of breast cancer [43].

Several studies have examined the presence of TNF-α^−308^G > A (rs1800629) in patients with different conditions, such as mortality, fatigue, postmenopausal status, and other symptoms after BC treatment [31, 33, 43]. Although these authors performed another study, their results support our finding that the TNF-α^−308^G > A (rs1800629) AA genotype and the A allele are associated with BC patients. These statistics either directly or indirectly corroborate our conclusions. Furthermore, a meta-analysis supported our results by showing that Asians [31] and Mexicans [52] had a greater frequency of the A allele, or AA genotype, which is highly engaged in BC.

Within the BC cohort, we investigated the association between TNF-α^−308^G > A (rs1800629) SNP and different tumor characteristics in BC patients. No risk has been estimated with different genetic models of TNF-α^−308^G > A (rs1800629) SNP in response to characteristics of tumors in breast cancer patients (Supplemental Tables 10–12). Moreover, when analyzing different prognostic models of BC, no risk estimates were noted for the different genetic models of TNF-α^−308^G > A (rs1800629) SNP in response to the poor prognostic model of the luminal **B model (ER**^**+ve**^**PR**^**+ve**^**Her2**^**+ve**^**)**, very poor prognostic model **(triple**^**–ve**^**)** or Her2-enriched poor model **(ER**^**−ve**^**PR**^**−ve**^**Her2**^**+ve**^**)** in contrast to the good prognostic status luminal A model **(ER**^**+ve**^**PR**^**+ve**^**Her2**^**−ve**^**)**. This may be because most BC patients harbor the GA genotype of the TNF-α-308G > A (rs1800629) SNP. The same observations have been documented for the Moroccan population [44].

The calculated NPIs among patients with different TNF-α^−308^G > A (rs1800629) genotypes were tested within different NPI groups (GPI, MPI, and PPI)**,** and significant increases were noted among patients with different TNF-α^−308^G > A (rs1800629) genotypes (GG > GA > AA) in the MPI group, where P = 0.01. Concerning hormonal status in BC patients, a significant rise in NPI was noted in patients with the AA genotype in positive hormones (ER or PR) compared to negative hormones. Additionally, for the GA genotype, a significant rise in the NPI was noted in Her2-positive and metastatic BC patients compared to negative patients. No study has examined this topic except for our former study of INF-γ + 874 T/A (rs2430561), in which we found no association with the calculated NPI between positive and negative hormone (ER or PR), Her2 protein expression or metastasis in BC patients [41].

As complex processes involving tumor spread, metastasis may occur along distant body parts from the original site. It is unknown what precise process causes BC metastasis to begin. Recently, it was discovered that cytokines were important mediators of BC metastasis. This information could aid in the creation of fresh treatment strategies to combat this issue [54, 55]. Moreover, TNF-α was discovered to be a useful cancer treatment tactic since it dramatically reduced tumor growth and stopped tumor spread [52, 56]. The involvement of the TNF-α gene SNP in BC metastasis has not been studied before. The current study revealed that the GA genotype of the TNF-α^−308^G > A (rs1800629) SNP was associated with more metastatic cases (21/24 cases) than the homozygote genotypes. Out of 21 metastatic BC patients, 13 presented MPI and 8 of them presented PPI. The GA genotype was also the only genotype associated with metastasis according to the TNBC prognosis model and Her2-enriched model **(ER**^**−ve**^**PR**^**−ve**^**Her2**^**+ve**^**)**; this may be due to the low number of patients in these models. However, the other models which include the good prognostic hormonal status **luminal A model, (ER**^**+ve**^**PR**^**+ve**^**Her2**^**−ve**^**)** and poor prognostic model of **luminal B model, (ER**^**+ve**^**PR**^**+ve**^**Her2**^**+ve**^**)** showed metastatic distributions concerning the different TNF-α^−308^G > A (rs1800629) genotypes.

## Conclusion

This is the first study to associate the functional TNF-α^−308^G > A (rs1800629) SNP, in Egyptian BC patients. The current investigation showed a substantial correlation between the TNF-α^−308^G > A (rs1800629) SNP and aggressive carcinogenesis in BC, indicating a potential function for the A allele and GA genotype in the pathophysiology of BC. This study validated the distribution of metastatic BC patients as well as the link between the A allele and a poor prognosis model and TNF-α^−308^G > A (rs1800629) SNP. The A allele and the GA genotype of the TNF-α^−308^G > A (rs1800629) SNP are strongly associated with breast cancer carcinogenesis, poor prognosis, and metastasis. It could be used as a valuable biomarker to guide the processes of increasing risk and carcinogenesis in breast cancer. The study’s limitations can be addressed by increasing the sample size to validate results and compare SNP with different tumor characteristics. Additionally, results should be distributed using multicentre samples.

## Supplementary Information

Below is the link to the electronic supplementary material.

Supplementary file1 (DOC 542 KB)

## Data Availability

The datasets generated during and/ or analyzed during the current study are available from the corresponding author upon reasonable request.

## Abbreviations

BC: Breast cancer
BBI: Benign breast inflammation
C: Controls
ARMS-PCR: Amplification refractory mutation system-polymerase chain reaction method
OR: Odds ratio
CI: 95% Confidence intervals
Her2: Human epidermal growth factor receptor 2
ER: Estrogen receptor
PR: Progesterone receptor
PAM50: Prediction analysis of microarray 50
INF-γ: Interferon-γ
TNF-α: Tumor necrosis factor-α
TGF-β: Transforming growth factor-β
IL: Interleukin
SNPs: Single-nucleotide polymorphisms
NK: Natural killer cells
Th1: T helper1
CD: Cluster of differentiation
IRB: Institutional review board
NPI: Nottingham prognostic index
GPI: Good prognostic index
MPI: Moderate prognostic index
PPI: Poor prognostic index
TNBC: Triple-negative BC
