# Supplementary material for: The functional TNF-α−308G > a single-nucleotide polymorphism (rs1800629): association with the predictive indices of breast cancer carcinogenesis
Source: Breast Cancer Res Treat. 2024 Nov 21;210(1):57–70. doi: 10.1007/s10549-024-07536-y (PMC11787156; doi:10.1007/s10549-024-07536-y)
Supplement: Supplementary file 1 — Supplementary file1 (DOC 542 KB) [file 10549_2024_7536_MOESM1_ESM.doc]

**The Functional TNF-α^-308^G>A Single Nucleotide Polymorphism (rs1800629): Association with the Predictive Indices of Breast Cancer Carcinogenesis**

**Sherif Refaat^a^, Hanan E. Al-Rashidi^b^, Rania A. Abd El Azeem^c^, Walaa E. Nouh^c^, Sahar Hamed^d*^ and Zeinab R. Attia^c^**

**Sherif Refaat^a^,** Department of Medical Oncology, Oncology Center, Mansoura University, Egypt. <https://orcid.org/0000-0002-9198-4900>.

[sherif_refaat2003@mans.edu.eg](mailto:sherif_refaat2003@mans.edu.eg)

**Hanan E. Al-Rashidi^b^,** Medical Laboratory Technology Department, College of Applied Medical Science, Taibah University, Madinah, Saudi Arabia.

[Hrashidi@taibahu.edu.sa](mailto:Hrashidi@taibahu.edu.sa)

**Rania A. Abd El Azeem^c^**, Mansoura University Children's Hospital, Mansoura University, Egypt. Second to Department of Clinical Laboratory Sciences, College of Medical Applied Sciences, University of Hafr Al Batin, Hafr Al Batin, Saudi Arabia.

[**http://orcid.org/0000-0002-8578-6537**](http://orcid.org/0000-0002-8578-6537)**.**

[**ramohmed@uhb.edu.sa**](mailto:ramohmed@uhb.edu.sa)

**Walaa E. Nouh^c^**, Mansoura University Children's Hospital, Mansoura University, Egypt**.** <https://orcid.org/0009-0006-2297-3956>**.**

[walaaesam78@mans.edu.eg](mailto:walaaesam78@mans.edu.eg)

**Sahar Hamed^d*^,** Urology and Nephrology Center, Mansoura University, Egypt**.** <https://orcid.org/0000-0002-6847-6553>.

[sahamed@mans.edu.eg](mailto:sahamed@mans.edu.eg)

**Zeinab R. Attia ^c^**, Mansoura University Children's Hospital, Mansoura University, Egypt**.** <https://orcid.org/0000-0003-1907-5419>.

[zeinabattia@mans.edu.eg](mailto:dr.dalia.tawfick@mans.edu.eg)

**Corresponding author:**

**Sahar Hamed^d*^, ^d*^**Urology and Nephrology Center, Mansoura University, Egypt**.** <https://orcid.org/0000-0002-6847-6553>.

[sahamed@mans.edu.eg](mailto:sahamed@mans.edu.eg)

**Table 1: Distribution of the TNF-α^-308^G>A (rs1800629) genotype frequencies in different tumor variants in the BC group (163 patients) in response to cancer stage**

| **Variables** | **GG^T1^** | **GG^T2^** | **GG^T3^** | **GG^T4^** | **AG^T1^** | **AG^T2^** | **AG^T3^** | **AG^T4^** | **AA^T2^** | **AA^T3^** |
| --- | --- | --- | --- | --- | --- | --- | --- | --- | --- | --- |
| **Stage** | 2 | 19 | 1 | 1 | 24 | 84 | 16 | 5 | 7 | 4 |
| **Node Status** | | | | | | | | | | |
| **56 (34.4) N0** | 2 | 6 | 0 | 0 | 13 | 27 | 5 | 1 | 2 | 0 |
| **42 (25.8) N1** | 0 | 4 | 0 | 1 | 9 | 21 | 3 | 1 | 2 | 1 |
| **40 (24.5) N2** | 0 | 7 | 1 | 0 | 2 | 18 | 6 | 1 | 3 | 2 |
| **25 (15.3) N3** | 0 | 2 | 0 | 0 | 0 | 18 | 2 | 2 | 0 | 1 |
| **Overall Grade** | | | | | | | | | | |
| **3 (1.8) G1** | 0 | 0 | 0 | 0 | 1 | 0 | 0 | 0 | 2 | 0 |
| **116 (71.2) G2** | 2 | 13 | 0 | 1 | 16 | 65 | 10 | 2 | 4 | 3 |
| **44 (27) G3** | 0 | 6 | 1 | 0 | 7 | 19 | 6 | 3 | 1 | 1 |
| **Tumor Size** | | | | | | | | | | |
| **14 (8.6) <2cm** | 1 | 0 | 0 | 0 | 12 | 1 | 0 | 0 | 0 | 0 |
| **121 (74.2) 2- 5 cm** | 1 | 18 | 0 | 0 | 10 | 80 | 2 | 2 | 7 | 1 |
| **28 (17.2) >5 cm** | 0 | 1 | 1 | 1 | 2 | 3 | 14 | 3 | 0 | 3 |
| **Nottingham Prognostic Index (NPI)** | | | | | | | | | | |
| **9 (5.5) >2.4- 3.4** | 2 | 0 | 0 | 0 | 6 | 0 | 0 | 0 | 1 | 0 |
| **121 (74.2) >3.4- 5.4** | 0 | 13 | 0 | 1 | 18 | 67 | 12 | 1 | 6 | 3 |
| **33 (20.3) >5.4** | 0 | 6 | 1 | 0 | 0 | 17 | 4 | 4 | 0 | 1 |
| **Estrogen receptor (ER)** | | | | | | | | | | |
| **33 (20.2) Negative** | 0 | 2 | 0 | 0 | 5 | 16 | 4 | 2 | 4 | 0 |
| **130 (79.8) Positive** | 2 | 17 | 1 | 1 | 19 | 68 | 12 | 3 | 3 | 4 |
| **Progesterone receptor (PR)** | | | | | | | | | | |
| **39 (23.9) Negative** | 0 | 3 | 0 | 0 | 2 | 21 | 7 | 4 | 2 | 0 |
| **124 (76.1) Positive** | 2 | 16 | 1 | 1 | 22 | 63 | 9 | 1 | 5 | 4 |
| **Her2/neu Expression** | | | | | | | | | | |
| **89 (54.6) Negative** | 1 | 10 | 0 | 0 | 14 | 47 | 4 | 4 | 5 | 2 |
| **74 (45.4) Positive** | 1 | 9 | 1 | 1 | 10 | 37 | 12 | 1 | 2 | 2 |
| **Metastasis** | | | | | | | | | | |
| **139 (85.3) Negative** | 2 | 18 | 1 | 1 | 23 | 71 | 11 | 3 | 5 | 4 |
| **24 (14.7) Positive** | 0 | 1 | 0 | 0 | 1 | 13 | 5 | 2 | 2 | 0 |
| **Operation Site** | | | | | | | | | | |
| **100 (61.4) Lt MRM** | 1 | 12 | 1 | 1 | 14 | 50 | 10 | 4 | 5 | 2 |
| **63 (38.6) Rt MRM** | 1 | 7 | 0 | 0 | 10 | 34 | 6 | 1 | 2 | 2 |
| **Age (year)** | | | | | | | | | | |
| **73 (44.8) < 50y** | 1 | 9 | 1 | 1 | 9 | 38 | 6 | 3 | 3 | 2 |
| **90 (55.2) > 50y** | 1 | 10 | 0 | 0 | 15 | 46 | 10 | 2 | 4 | 2 |

**Table 2: Distribution of the TNF-α^-308^G>A (rs1800629) genotype frequencies in different tumor variants in the BC group (163 patients) in response to tumor grade**

| **Variables** | **GG^G2^** | **GG^G3^** | **AG^G1^** | **AG^G2^** | **AG^G3^** | **AA^G1^** | **AA^G2^** | **AA^G3^** |
| --- | --- | --- | --- | --- | --- | --- | --- | --- |
| **Tumor Grade** | 16 | 7 | 1 | 93 | 35 | 2 | 7 | 2 |
| **Cancer Stage** | | | | | | | | |
| **26 (15.9) T1** | 2 | 0 | 1 | 16 | 7 | 0 | 0 | 0 |
| **110 (67.5) T2** | 13 | 6 | 0 | 65 | 19 | 2 | 4 | 1 |
| **21 (12.9) T3** | 0 | 1 | 0 | 10 | 6 | 0 | 3 | 1 |
| **6 (3.7) T4** | 1 | 0 | 0 | 2 | 3 | 0 | 0 | 0 |
| **Node Status** | | | | | | | | |
| **56 (34.4) N0** | 8 | 0 | 1 | 33 | 12 | 1 | 0 | 1 |
| **42 (25.8) N1** | 3 | 2 | 0 | 27 | 7 | 0 | 2 | 1 |
| **40 (24.5) N2** | 5 | 3 | 0 | 19 | 8 | 1 | 4 | 0 |
| **25 (15.3) N3** | 0 | 2 | 0 | 14 | 8 | 0 | 1 | 0 |
| **Tumor Size** | | | | | | | | |
| **14 (8.6) <2cm** | 1 | 0 | 0 | 9 | 4 | 0 | 0 | 0 |
| **121 (74.2) 2- 5 cm** | 13 | 6 | 1 | 70 | 23 | 2 | 5 | 1 |
| **28 (17.2) >5 cm** | 2 | 1 | 0 | 14 | 8 | 0 | 2 | 1 |
| **Nottingham Prognostic Index (NPI)** | | | | | | | | |
| **9 (5.5) >2.4- 3.4** | 2 | 0 | 1 | 5 | 0 | 1 | 0 | 0 |
| **121 (74.2) >3.4- 5.4** | 14 | 0 | 0 | 85 | 13 | 1 | 7 | 1 |
| **33 (20.3) >5.4** | 0 | 7 | 0 | 3 | 22 | 0 | 0 | 1 |
| **Estrogen receptor (ER)** | | | | | | | | |
| **33 (20.2) Negative** | 2 | 0 | 0 | 20 | 7 | 2 | 1 | 1 |
| **130 (79.8) Positive** | 14 | 7 | 1 | 73 | 28 | 0 | 6 | 1 |
| **Progesterone receptor (PR)** | | | | | | | | |
| **39 (23.9) Negative** | 3 | 0 | 0 | 22 | 12 | 2 | 0 | 0 |
| **124 (76.1) Positive** | 13 | 7 | 1 | 71 | 23 | 0 | 7 | 2 |
| **Her2/neu Expression** | | | | | | | | |
| **89 (54.6) Negative** | 10 | 3 | 1 | 54 | 14 | 1 | 5 | 1 |
| **74 (45.4) Positive** | 6 | 4 |  | 39 | 21 | 1 | 2 | 1 |
| **Metastasis** | | | | | | | | |
| **139 (85.3) Negative** | 16 | 6 | 1 | 82 | 25 | 2 | 5 | 2 |
| **24 (14.7) Positive** | 0 | 1 | 0 | 11 | 10 | 0 | 2 | 0 |
| **Operation Site** | | | | | | | | |
| **100 (61.4) Lt MRM** | 11 | 4 |  | 59 | 19 | 1 | 5 | 1 |
| **63 (38.6) Rt MRM** | 5 | 3 | 1 | 34 | 16 | 1 | 2 | 1 |
| **Age (year)** | | | | | | | | |
| **73 (44.8) < 50y** | 10 | 2 | 0 | 39 | 17 | 1 | 4 | 0 |
| **90 (55.2) > 50y** | 6 | 5 | 1 | 54 | 18 | 1 | 3 | 2 |

**Table 3: Distribution of the TNF-α^-308^G>A (rs1800629) genotype frequencies in different tumor variants in the BC group (163 patients) in response to tumor size**

| **Variables** | **GG^S^** | **GG^M^** | **GG^L^** | **AG^S^** | **AG^M^** | **AG^L^** | **AA^M^** | **AA^L^** |
| --- | --- | --- | --- | --- | --- | --- | --- | --- |
| **Tumor Size** | 1 | 19 | 3 | 13 | 94 | 22 | 8 | 3 |
| **Cancer Stage** | | | | | | | | |
| **26 (15.9) T1** | 1 | 1 | 0 | 12 | 10 | 2 | 0 | 0 |
| **110 (67.5) T2** | 0 | 18 | 1 | 1 | 80 | 3 | 7 | 0 |
| **21 (12.9) T3** | 0 | 0 | 1 | 0 | 2 | 14 | 1 | 3 |
| **6 (3.7) T4** | 0 | 0 | 1 | 0 | 2 | 3 | 0 | 0 |
| **Node Status** | | | | | | | | |
| **56 (34.4) N0** | 1 | 7 | 0 | 6 | 33 | 7 | 2 | 0 |
| **42 (25.8) N1** | 0 | 4 | 1 | 6 | 24 | 4 | 2 | 1 |
| **40 (24.5) N2** | 0 | 6 | 2 | 0 | 20 | 7 | 3 | 2 |
| **25 (15.3) N3** | 0 | 2 | 0 | 1 | 17 | 4 | 1 | 0 |
| **Overall Grade** | | | | | | | | |
| **3 (1.8) G1** | 0 | 0 | 0 | 0 | 1 | 0 | 2 | 0 |
| **116 (71.2) G2** | 1 | 13 | 2 | 9 | 70 | 14 | 5 | 2 |
| **44 (27) G3** | 0 | 6 | 1 | 4 | 23 | 8 | 1 | 1 |
| **Nottingham Prognostic Index (NPI)** | | | | | | | | |
| **9 (5.5) >2.4- 3.4** | 1 | 1 | 0 | 4 | 2 | 0 | 1 | 0 |
| **121 (74.2) >3.4- 5.4** | 0 | 12 | 2 | 8 | 76 | 14 | 7 | 2 |
| **33 (20.3) >5.4** | 0 | 6 | 1 | 1 | 16 | 8 | 0 | 1 |
| **Estrogen receptor (ER)** | | | | | | | | |
| **33 (20.2) Negative** | 0 | 2 | 0 | 2 | 20 | 5 | 4 | 0 |
| **130 (79.8) Positive** | 1 | 17 | 3 | 11 | 74 | 17 | 4 | 3 |
| **Progesterone receptor (PR)** | | | | | | | | |
| **39 (23.9) Negative** | 0 | 3 | 0 | 1 | 22 | 11 | 2 | 0 |
| **124 (76.1) Positive** | 1 | 16 | 3 | 12 | 72 | 11 | 6 | 3 |
| **Her2/neu Expression** | | | | | | | | |
| **89 (54.6) Negative** | 1 | 9 | 3 | 10 | 49 | 10 | 6 | 1 |
| **74 (45.4) Positive** | 0 | 10 | 0 | 3 | 45 | 12 | 2 | 2 |
| **Metastasis** | | | | | | | | |
| **139 (85.3) Negative** | 1 | 18 | 3 | 11 | 82 | 15 | 6 | 3 |
| **24 (14.7) Positive** | 0 | 1 | 0 | 2 | 12 | 7 | 2 | 0 |
| **Operation Site** | | | | | | | | |
| **100 (61.4) Lt MRM** | 0 | 13 | 2 | 8 | 54 | 16 | 5 | 2 |
| **63 (38.6) Rt MRM** | 1 | 6 | 1 | 5 | 40 | 6 | 3 | 1 |
| **Age (year)** | | | | | | | | |
| **73 (44.8) < 50y** | 0 | 9 | 0 | 5 | 42 | 9 | 3 | 2 |
| **90 (55.2) > 50y** | 1 | 10 | 3 | 8 | 52 | 13 | 5 | 1 |

**S=<2cm; M= 2- 5 cm; L= >5 cm.**

**Table 4: Distribution of the TNF-α^-308^G>A (rs1800629) genotype frequencies in different tumor variants in the BC group (163 patients) in response to estrogen receptor (ER)**

| **Variables** | **GG^-ve^** | **GG^+ve^** | **AG^-ve^** | **AG^+ve^** | **AA^-ve^** | **AA^+ve^** |
| --- | --- | --- | --- | --- | --- | --- |
| **ER** | 2 | 21 | 27 | 102 | 4 | 7 |
| **Cancer Stage** | | | | | | |
| **26 (15.9) T1** | 0 | 2 | 5 | 19 | 0 | 0 |
| **110 (67.5) T2** | 2 | 17 | 16 | 68 | 4 | 3 |
| **21 (12.9) T3** | 0 | 1 | 4 | 12 | 0 | 4 |
| **6 (3.7) T4** | 0 | 1 | 2 | 3 | 0 | 0 |
| **Node Status** | | | | | | |
| **56 (34.4) N0** | 1 | 7 | 10 | 36 | 2 | 0 |
| **42 (25.8) N1** | 0 | 5 | 5 | 29 | 1 | 2 |
| **40 (24.5) N2** | 1 | 7 | 5 | 22 | 1 | 4 |
| **25 (15.3) N3** | 0 | 2 | 7 | 15 | 0 | 1 |
| **Overall Grade** | | | | | | |
| **3 (1.8) G1** | 0 | 0 | 0 | 1 | 2 | 0 |
| **116 (71.2) G2** | 2 | 14 | 20 | 73 | 1 | 6 |
| **44 (27) G3** | 0 | 7 | 7 | 28 | 1 | 1 |
| **Tumor Size** | | | | | | |
| **14 (8.6) <2cm** | 0 | 1 | 2 | 11 | 0 | 0 |
| **121 (74.2) 2- 5 cm** | 2 | 17 | 20 | 74 | 4 | 6 |
| **28 (17.2) >5 cm** | 0 | 3 | 5 | 17 | 0 | 1 |
| **Nottingham Prognostic Index (NPI)** | | | | | | |
| **9 (5.5) >2.4- 3.4** | 0 | 2 | 2 | 4 | 1 | 0 |
| **121 (74.2) >3.4- 5.4** | 2 | 2 | 19 | 79 | 3 | 6 |
| **33 (20.3) >5.4** | 0 | 7 | 6 | 19 | 0 | 1 |
| **Progesterone receptor (PR)** | | | | | | |
| **39 (23.9) Negative** | 2 | 1 | 20 | 14 | 2 | 0 |
| **124 (76.1) Positive** | 0 | 20 | 7 | 88 | 2 | 7 |
| **Her2/neu Expression** | | | | | | |
| **89 (54.6) Negative** | 0 | 13 | 13 | 56 | 3 | 0 |
| **74 (45.4) Positive** | 2 | 8 | 14 | 46 | 1 | 7 |
| **Metastasis** | | | | | | |
| **139 (85.3) Negative** | 2 | 20 | 24 | 84 | 4 | 5 |
| **24 (14.7) Positive** | 0 | 1 | 3 | 18 | 0 | 2 |
| **Operation Site** | | | | | | |
| **100 (61.4) Lt MRM** | 1 | 14 | 19 | 59 | 2 | 5 |
| **63 (38.6) Rt MRM** | 1 | 7 | 8 | 43 | 2 | 2 |
| **Age (year)** | | | | | | |
| **73 (44.8) < 50y** | 1 | 11 | 12 | 44 | 1 | 4 |
| **90 (55.2) > 50y** | 1 | 10 | 15 | 58 | 3 | 3 |

**Table 5: Distribution of the TNF-α^-308^G>A (rs1800629) genotype frequencies in different tumor variants in the BC group (163 patients) in response to progesterone receptor (PR)**

| **Variables** | **GG^-ve^** | **GG^+ve^** | **AG^-ve^** | **AG^+ve^** | **AA^-ve^** | **AA^+ve^** |
| --- | --- | --- | --- | --- | --- | --- |
| **PR** | 3 | 20 | 34 | 95 | 2 | 9 |
| **Cancer Stage** | | | | | | |
| **26 (15.9) T1** | 0 | 2 | 2 | 22 | 0 | 0 |
| **110 (67.5) T2** | 3 | 16 | 21 | 63 | 2 | 5 |
| **21 (12.9) T3** | 0 | 1 | 7 | 9 | 0 | 4 |
| **6 (3.7) T4** | 0 | 1 | 4 | 1 | 0 | 0 |
| **Node Status** | | | | | | |
| **56 (34.4) N0** | 2 | 6 | 12 | 34 | 1 | 1 |
| **42 (25.8) N1** | 0 | 5 | 5 | 29 | 0 | 3 |
| **40 (24.5) N2** | 1 | 7 | 7 | 20 | 1 | 4 |
| **25 (15.3) N3** | 0 | 2 | 10 | 12 | 0 | 1 |
| **Overall Grade** | | | | | | |
| **3 (1.8) G1** | 0 | 0 | 1 | 1 | 2 | 0 |
| **116 (71.2) G2** | 3 | 13 | 22 | 71 | 0 | 7 |
| **44 (27) G3** | 0 | 7 | 12 | 23 | 0 | 2 |
| **Tumor Size** | | | | | | |
| **14 (8.6) <2cm** | 0 | 1 | 1 | 12 | 0 | 0 |
| **121 (74.2) 2- 5 cm** | 3 | 16 | 22 | 72 | 2 | 6 |
| **28 (17.2) >5 cm** | 0 | 3 | 11 | 11 | 0 | 3 |
| **Nottingham Prognostic Index (NPI)** | | | | | | |
| **9 (5.5) >2.4- 3.4** | 0 | 2 | 0 | 6 | 1 | 0 |
| **121 (74.2) >3.4- 5.4** | 3 | 11 | 24 | 74 | 1 | 8 |
| **33 (20.3) >5.4** | 0 | 7 | 10 | 15 | 0 | 1 |
| **Estrogen receptor (ER)** | | | | | | |
| **33 (20.2) Negative** | 2 | 0 | 20 | 7 | 2 | 2 |
| **130 (79.8) Positive** | 1 | 20 | 14 | 88 | 0 | 7 |
| **Her2/neu Expression** | | | | | | |
| **89 (54.6) Negative** | 1 | 12 | 18 | 51 | 1 | 6 |
| **74 (45.4) Positive** | 2 | 8 | 16 | 44 | 1 | 3 |
| **Metastasis** | | | | | | |
| **139 (85.3) Negative** | 3 | 19 | 26 | 82 | 2 | 7 |
| **24 (14.7) Positive** | 0 | 0 | 8 | 13 | 0 | 2 |
| **Operation Site** | | | | | | |
| **100 (61.4) Lt MRM** | 2 | 13 | 24 | 54 | 1 | 6 |
| **63 (38.6) Rt MRM** | 1 | 7 | 10 | 41 | 1 | 3 |
| **Age (year)** | | | | | | |
| **73 (44.8) < 50y** | 2 | 10 | 14 | 42 | 1 | 4 |
| **90 (55.2) > 50y** | 1 | 10 | 20 | 53 | 1 | 5 |

**Table 6: Distribution of the TNF-α^-308^G>A (rs1800629) genotype frequencies in different tumor variants in the BC group (163 patients) in response to Her2/neu expression**

| **Variables** | **GG^-ve^** | **GG^+ve^** | **AG^-ve^** | **AG^+ve^** | **AA^-ve^** | **AA^+ve^** |
| --- | --- | --- | --- | --- | --- | --- |
| **Her2/neu** | 13 | 10 | 69 | 60 | 7 | 4 |
| **Cancer Stage** | | | | | | |
| **26 (15.9) T1** | 1 | 1 | 14 | 10 | 0 | 0 |
| **110 (67.5) T2** | 10 | 9 | 47 | 37 | 5 | 2 |
| **21 (12.9) T3** | 1 | 0 | 4 | 12 | 2 | 2 |
| **6 (3.7) T4** | 1 | 0 | 4 | 1 | 0 | 0 |
| **Node Status** | | | | | | |
| **56 (34.4) N0** | 5 | 3 | 29 | 17 | 2 | 0 |
| **42 (25.8) N1** | 4 | 1 | 19 | 15 | 2 | 1 |
| **40 (24.5) N2** | 3 | 5 | 12 | 15 | 2 | 3 |
| **25 (15.3) N3** | 1 | 1 | 9 | 13 | 1 | 0 |
| **Overall Grade** | | | | | | |
| **3 (1.8) G1** | 0 | 0 | 1 | 0 | 1 | 1 |
| **116 (71.2) G2** | 10 | 6 | 54 | 39 | 5 | 2 |
| **44 (27) G3** | 3 | 4 | 14 | 21 | 1 | 1 |
| **Tumor Size** | | | | | | |
| **14 (8.6) <2cm** | 1 | 10 | 10 | 3 | 0 | 0 |
| **121 (74.2) 2- 5 cm** | 9 | 0 | 49 | 45 | 6 | 2 |
| **28 (17.2) >5 cm** | 3 | 0 | 10 | 12 | 1 | 2 |
| **Nottingham Prognostic Index (NPI)** | | | | | | |
| **9 (5.5) >2.4- 3.4** | 1 | 1 | 3 | 3 | 1 | 0 |
| **121 (74.2) >3.4- 5.4** | 9 | 5 | 56 | 42 | 6 | 3 |
| **33 (20.3) >5.4** | 3 | 4 | 10 | 15 | 0 | 1 |
| **Estrogen receptor (ER)** | | | | | | |
| **33 (20.2) Negative** | 0 | 2 | 13 | 14 | 3 | 1 |
| **130 (79.8) Positive** | 13 | 8 | 56 | 46 | 4 | 4 |
| **Progesterone receptor (PR)** | | | | | | |
| **39 (23.9) Negative** | 1 | 2 | 18 | 16 | 1 | 1 |
| **124 (76.1) Positive** | 12 | 8 | 51 | 44 | 6 | 4 |
| **Metastasis** | | | | | | |
| **139 (85.3) Negative** | 12 | 10 | 59 | 49 | 6 | 3 |
| **24 (14.7) Positive** | 1 | 0 | 10 | 11 | 1 | 1 |
| **Operation Site** | | | | | | |
| **100 (61.4) Lt MRM** | 8 | 7 | 42 | 36 | 3 | 0 |
| **63 (38.6) Rt MRM** | 5 | 3 | 27 | 24 | 4 | 4 |
| **Age (year)** | | | | | | |
| **73 (44.8) < 50y** | 7 | 5 | 29 | 27 | 2 | 3 |
| **90 (55.2) > 50y** | 6 | 5 | 40 | 33 | 5 | 1 |

**Table 7: Distribution of the TNF-α^-308^G>A (rs1800629) genotype frequencies in different tumor variants in the BC group (163 patients) in response to metastasis**

| **Variables** | **GG^-ve^** | **GG^+ve^** | **AG^-ve^** | **AG^+ve^** | **AA^-ve^** | **AA^+ve^** |
| --- | --- | --- | --- | --- | --- | --- |
| **Metastasis** | 22 | 1 | 108 | 21 | 9 | 2 |
| **Cancer Stage** | | | | | | |
| **26 (15.9) T1** | 2 | 0 | 23 | 1 | 0 | 0 |
| **110 (67.5) T2** | 18 | 1 | 71 | 13 | 5 | 2 |
| **21 (12.9) T3** | 1 | 0 | 11 | 5 | 4 | 0 |
| **6 (3.7) T4** | 1 | 0 | 3 | 2 | 0 | 0 |
| **Node Status** | | | | | | |
| **56 (34.4) N0** | 8 | 0 | 40 | 6 | 2 | 0 |
| **42 (25.8) N1** | 4 | 1 | 28 | 6 | 2 | 1 |
| **40 (24.5) N2** | 8 | 0 | 23 | 4 | 4 | 1 |
| **25 (15.3) N3** | 2 | 0 | 17 | 5 | 1 | 0 |
| **Overall Grade** | | | | | | |
| **3 (1.8) G1** | 0 | 0 | 1 | 0 | 2 | 0 |
| **116 (71.2) G2** | 16 | 0 | 82 | 11 | 5 | 2 |
| **44 (27) G3** | 6 | 1 | 25 | 10 | 2 | 0 |
| **Tumor Size** | | | | | | |
| **14 (8.6) <2cm** | 1 | 0 | 11 | 2 | 0 | 0 |
| **121 (74.2) 2- 5 cm** | 18 | 1 | 82 | 12 | 6 | 2 |
| **28 (17.2) >5 cm** | 3 | 0 | 15 | 7 | 3 | 0 |
| **Nottingham Prognostic Index (NPI)** | | | | | | |
| **9 (5.5) >2.4- 3.4** | 2 | 0 | 6 | 0 | 1 | 0 |
| **121 (74.2) >3.4- 5.4** | 14 | 0 | 85 | 13 | 7 | 2 |
| **33 (20.3) >5.4** | 6 | 1 | 17 | 8 | 1 | 0 |
| **Estrogen receptor (ER)** | | | | | | |
| **33 (20.2) Negative** | 2 | 0 | 24 | 3 | 4 | 0 |
| **130 (79.8) Positive** | 20 | 1 | 84 | 18 | 5 | 2 |
| **Progesterone receptor (PR)** | | | | | | |
| **39 (23.9) Negative** | 3 | 0 | 26 | 8 | 2 | 0 |
| **124 (76.1) Positive** | 19 | 1 | 82 | 13 | 7 | 2 |
| **Her2/neu Expression** | | | | | | |
| **89 (54.6) Negative** | 12 | 1 | 59 | 10 | 6 | 1 |
| **74 (45.4) Positive** | 10 | 0 | 49 | 11 | 3 | 1 |
| **Operation Site** | | | | | | |
| **100 (61.4) Lt MRM** | 4 | 1 | 64 | 14 | 5 | 2 |
| **63 (38.6) Rt MRM** | 8 | 0 | 44 | 7 | 4 | 0 |
| **Age (year)** | | | | | | |
| **73 (44.8) < 50y** | 12 | 0 | 46 | 10 | 4 | 1 |
| **90 (55.2) > 50y** | 10 | 1 | 62 | 11 | 5 | 1 |

**Table 8: Distribution of the TNF-α^-308^G>A (rs1800629) genotype frequencies in different tumor variants in the BC group (163 patients) in response to patient’s operation site**

| **Variables** | **GG^Lt^** | **GG^Rt^** | **AG^Lt^** | **AG^Rt^** | **AA^Lt^** | **AA^Rt^** |
| --- | --- | --- | --- | --- | --- | --- |
| **Operation Site** | 15 | 8 | 78 | 51 | 7 | 4 |
| **Cancer Stage** | | | | | | |
| **26 (15.9) T1** | 1 | 1 | 14 | 10 | 0 | 0 |
| **110 (67.5) T2** | 12 | 7 | 50 | 34 | 5 | 2 |
| **21 (12.9) T3** | 1 | 0 | 10 | 6 | 2 | 2 |
| **6 (3.7) T4** | 1 | 0 | 4 | 1 | 0 | 0 |
| **Node Status** | | | | | | |
| **56 (34.4) N0** | 6 | 2 | 28 | 18 | 0 | 2 |
| **42 (25.8) N1** | 4 | 1 | 21 | 13 | 3 | 0 |
| **40 (24.5) N2** | 4 | 4 | 17 | 10 | 4 | 1 |
| **25 (15.3) N3** | 1 | 1 | 12 | 10 | 0 | 1 |
| **Overall Grade** | | | | | | |
| **3 (1.8) G1** | 0 | 0 | 0 | 1 | 1 | 1 |
| **116 (71.2) G2** | 11 | 5 | 59 | 34 | 5 | 2 |
| **44 (27) G3** | 4 | 3 | 19 | 16 | 1 | 1 |
| **Tumor Size** | | | | | | |
| **14 (8.6) <2cm** | 0 | 1 | 8 | 5 | 0 | 0 |
| **121 (74.2) 2- 5 cm** | 13 | 6 | 54 | 40 | 5 | 3 |
| **28 (17.2) >5 cm** | 2 | 1 | 16 | 6 | 2 | 1 |
| **Nottingham Prognostic Index (NPI)** | | | | | | |
| **9 (5.5) >2.4- 3.4** | 1 | 1 | 3 | 3 | 0 | 1 |
| **121 (74.2) >3.4- 5.4** | 10 | 4 | 61 | 37 | 6 | 4 |
| **33 (20.3) >5.4** | 4 | 3 | 14 | 11 | 1 | 0 |
| **Estrogen receptor (ER)** | | | | | | |
| **33 (20.2) Negative** | 1 | 1 | 19 | 8 | 2 | 2 |
| **130 (79.8) Positive** | 14 | 7 | 59 | 43 | 5 | 2 |
| **Progesterone receptor (PR)** | | | | | | |
| **39 (23.9) Negative** | 2 | 1 | 24 | 10 | 1 | 1 |
| **124 (76.1) Positive** | 13 | 7 | 54 | 41 | 6 | 3 |
| **Her2/neu Expression** | | | | | | |
| **89 (54.6) Negative** | 8 | 5 | 42 | 24 | 3 | 4 |
| **74 (45.4) Positive** | 7 | 3 | 36 | 27 | 4 | 0 |
| **Metastasis** | | | | | | |
| **139 (85.3) Negative** | 14 | 8 | 64 | 44 | 5 | 4 |
| **24 (14.7) Positive** | 1 | 0 | 14 | 7 | 2 | 0 |
| **Age (year)** | | | | | | |
| **73 (44.8) < 50y** | 8 | 4 | 32 | 24 | 4 | 1 |
| **90 (55.2) > 50y** | 7 | 4 | 46 | 27 | 3 | 3 |

**Table 9: Distribution of the TNF-α^-308^G>A (rs1800629) genotype frequencies in different tumor variants in the BC group (163 patients) in response to patients’ age**

| **Variables** | **GG^<50^** | **GG^>50^** | **AG^<50^** | **AG^>50^** | **AA^<50^** | **AA^>50^** |
| --- | --- | --- | --- | --- | --- | --- |
| **Age** | 12 | 11 | 56 | 73 | 5 | 6 |
| **Cancer Stage** | | | | | | |
| **26 (15.9) T1** | 1 | 1 | 9 | 15 | 0 | 0 |
| **110 (67.5) T2** | 9 | 10 | 38 | 46 | 3 | 4 |
| **21 (12.9) T3** | 1 | 0 | 6 | 10 | 2 | 2 |
| **6 (3.7) T4** | 1 | 0 | 3 | 2 | 0 | 1 |
| **Node Status** | | | | | | |
| **56 (34.4) N0** | 4 | 4 | 21 | 25 | 0 | 2 |
| **42 (25.8) N1** | 2 | 3 | 10 | 24 | 0 | 3 |
| **40 (24.5) N2** | 6 | 2 | 16 | 11 | 5 | 0 |
| **25 (15.3) N3** | 0 | 2 | 9 | 13 | 0 | 1 |
| **Overall Grade** | | | | | | |
| **3 (1.8) G1** | 0 | 0 | 0 | 1 | 1 | 1 |
| **116 (71.2) G2** | 10 | 6 | 39 | 54 | 4 | 3 |
| **44 (27) G3** | 2 | 5 | 17 | 18 | 0 | 2 |
| **Tumor Size** | | | | | | |
| **14 (8.6) <2cm** | 0 | 1 | 5 | 8 | 0 | 0 |
| **121 (74.2) 2- 5 cm** | 9 | 10 | 42 | 52 | 3 | 5 |
| **28 (17.2) >5 cm** | 3 | 0 | 9 | 13 | 2 | 1 |
| **Nottingham Prognostic Index (NPI)** | | | | | | |
| **9 (5.5) >2.4- 3.4** | 1 | 1 | 3 | 3 | 0 | 1 |
| **121 (74.2) >3.4- 5.4** | 9 | 5 | 42 | 56 | 5 | 4 |
| **33 (20.3) >5.4** | 2 | 5 | 11 | 14 | 0 | 1 |
| **Estrogen receptor (ER)** | | | | | | |
| **33 (20.2) Negative** | 1 | 1 | 12 | 15 | 1 | 3 |
| **130 (79.8) Positive** | 11 | 10 | 44 | 58 | 4 | 3 |
| **Progesterone receptor (PR)** | | | | | | |
| **39 (23.9) Negative** | 2 | 1 | 14 | 20 | 1 | 1 |
| **124 (76.1) Positive** | 10 | 10 | 42 | 53 | 4 | 5 |
| **Her2/neu Expression** | | | | | | |
| **89 (54.6) Negative** | 7 | 6 | 29 | 40 | 2 | 5 |
| **74 (45.4) Positive** | 5 | 5 | 27 | 33 | 3 | 1 |
| **Metastasis** | | | | | | |
| **139 (85.3) Negative** | 12 | 10 | 46 | 62 | 4 | 5 |
| **24 (14.7) Positive** | 0 | 1 | 10 | 11 | 1 | 1 |
| **Operation Site** | | | | | | |
| **100 (61.4) Lt MRM** | 8 | 7 | 32 | 46 | 4 | 3 |
| **63 (38.6) Rt MRM** | 4 | 4 | 24 | 27 | 1 | 3 |

**Table 10: Distribution of the TNF-α^-308^G>A (rs1800629) genotype frequencies with risk estimate in response to hormonal markers; estrogen receptor (ER) and progesterone receptor (PR) in the BC group**

| **Estrogen receptor (ER)** | | | | |
| --- | --- | --- | --- | --- |
| **Model** | **Genotype # (%)** | | **OR (95% CI)** | **P** |
| **Codominant** | **Negative 33 (20.3)** | **Positive 130 (79.7)** |  |  |
| **GG** | 2 (6.1) | 21 (16.1) | 1 |  |
| **GA** | 27 (81.8) | 102 (78.5) | 0.36 (0.08- 1.63) | 0.13 |
| **AA** | 4 (12.1) | 7 (5.4) | 0.17 (0.02- 1.11) | 0.07 |
| **Dominant** | AA + GA **vs GG** | | 0.34 (0.07- 1.50) | 0.11 |
| **Recessive** | **AA vs** GG + GA | | 2.42 (0.66- 8.83) | 0.16 |
| **Overdominant** | **GA vs** AA + GG | | 1.23 (0.46- 3.28) | 0.44 |
| **Progesterone receptor (PR)** | | | | |
| **Model** | **Genotype # (%)** | | **OR (95% CI)** | **P** |
| **Codominant** | **Negative 39 (23.9)** | **Positive 124 (76.1)** |  |  |
| **GG** | 3 (7.7) | 20 (16.1) | 1 |  |
| **GA** | 34 (87.2) | 95 (76.6) | 0.42 (0.12- 1.5) | 0.13 |
| **AA** | 2 (5.1) | 9 (7.3) | 0.67 (0.09- 4.76) | 0.53 |
| **Dominant** | AA + GA **vs GG** | | 0.43 (0.12- 1.54) | 0.14 |
| **Recessive** | **AA vs** GG + GA | | 0.69 (0.14- 3.34) | 0.48 |
| **Overdominant** | **GA vs** AA + GG | | 2.07 (0.74- 5.79) | 0.11 |

**Table 11: Distribution of the TNF-α^-308^G>A (rs1800629) genotype frequencies with risk estimate in response to Her2/neu protein expression marker and metastasis status in the BC group**

| **Her2/neu expression marker** | | | | | | | |
| --- | --- | --- | --- | --- | --- | --- | --- |
| **Model** | **Genotype # (%)** | | | **OR (95% CI)** | | **P** | |
| **Codominant** | **Negative 89 (54.6)** | **Positive 74 (45.4)** | |  | |  | |
| **GG** | 13 (14.6) | 10 (13.5) | | 1 | |  | |
| **GA** | 69 (77.5) | 60 (81.1) | | 1.13 (0.46- 2.76) | | 0.48 | |
| **AA** | 7 (7.9) | 4 (5.4) | | 0.74 (0.17- 3.26) | | 0.49 | |
| **Dominant** | AA + GA **vs GG** | | | 1.09 (0.45- 2.66) | | 0.51 | |
| **Recessive** | **AA vs** GG + GA | | | 1.49 (0.42- 5.31) | | 0.38 | |
| **Overdominant** | **GA vs** AA + GG | | | 0.8 (0.37- 1.73) | | 0.36 | |
| **Metastasis** | | | | | | | |
| **Model** | **Genotype # (%)** | | | | **OR (95% CI)** | | **P** |
| **Codominant** | **Negative 139 (85.3)** | | **Positive 24 (14.7)** | |  | |  |
| **GG** | 22 (15.8) | | 1 (4.2) | | 1 | |  |
| **GA** | 108 (77.7) | | 21 (87.5) | | 4.28 (0.54- 33.49) | | 0.11 |
| **AA** | 9 (6.5) | | 2 (8.3) | | 4.89 (0.39- 60.92) | | 0.23 |
| **Dominant** | AA + GA **vs GG** | | | | 4.32 (0.24- 2.4) | | 0.10 |
| **Recessive** | **AA vs** GG + GA | | | | 0.76 (0.55- 33.7) | | 0.5 |
| **Overdominant** | **GA vs** AA + GG | | | | 0.49 (0.14- 1. 78) | | 0.21 |

**Table 12: Distribution of the TNF-α^-308^G>A (rs1800629) genotype frequencies with risk estimate in response to operation type and age (years) in the BC group**

| **Operation Type** | | | | | | | |
| --- | --- | --- | --- | --- | --- | --- | --- |
| **Model** | **Genotype # (%)** | | | | **OR (95% CI)** | **P** | |
| **Codominant** | **Lt MRM 100 (61.4)** | **Rt MRM 63 (38.6)** | | |  |  | |
| **GG** | 15 (15) | 8 (12.7) | | | 1 |  | |
| **GA** | 78 (78) | 51 (81) | | | 1.22 (0.48- 3.1) | 0.42 | |
| **AA** | 7 (7) | 4 (6.3) | | | 1.07 (0.24- 4.79) | 0.61 | |
| **Dominant** | AA + GA **vs GG** | | | | 1.21 (0.48- 3.05) | 0.43 | |
| **Recessive** | **AA vs** GG + GA | | | | 1.09 (0.30- 3.89) | 0.58 | |
| **Overdominant** | **GA vs** AA + GG | | | | 0.83 (0.38- 1.83) | 0.4 | |
| **Age (years)** | | | | | | | |
| **Model** | **Genotype # (%)** | | | **OR (95% CI)** | | | **P** |
| **Codominant** | **< 50y 73 (44.8)** | | **> 50y 90 (55.2)** |  | | |  |
| **GG** | 12 (16.4) | | 11 (12.2) | 1 | | |  |
| **GA** | 56 (76.7) | | 73 (81.1) | 1.42 (0.58- 3.46) | | | 0.29 |
| **AA** | 5 (6.8) | | 6 (6.7) | 1.31 (0.31- 5.53) | | | 0.5 |
| **Dominant** | AA + GA **vs GG** | | | 1.41 (0.58- 3.42) | | | 0.29 |
| **Recessive** | **AA vs** GG + GA | | | 1.03 (0.3- 3.52) | | | 0.6 |
| **Overdominant** | **GA vs** AA + GG | | | 0.76 (0.36- 1.63) | | | 0.31 |

**Table 13: Distribution of the TNF-α^-308^G>A (rs1800629) genotype frequencies with risk estimate in Triple^–ve^ (very poor prognostic model) vs very poor prognostic Her2-enriched model (ER^-ve^PR^-ve^Her2^+ve^) in the BC group**

| **Model** | **Genotype # (%)** | | **OR (95% CI)** | **P** |
| --- | --- | --- | --- | --- |
| **Codominant** | **ER^-ve^PR^-ve^Her2^+ve^ 14cases (Her2-enrich)** | **ER^-ve^PR^-ve^Her2^-ve^ 11 cases (triple^–ve^)** |  |  |
| **GG** | 2 (14.3) | 0 (0) | 1 |  |
| **GA** | 11 (78.6) | 10 (91) | 1.91 (1. 27- 2.87) | 0.3 |
| **AA** | 1 (7.1) | 1 (9) | 2.0 (0.5- 7.99) | 0.5 |
| **Dominant** | AA + GA **vs GG** | | 1.91 (1.29- 2.83) | 0.30 |
| **Recessive** | **AA vs** GG + GA | | 0.77 (0.04- 13.86) | 0.69 |
| **Overdominant** | **GA vs** AA + GG | | 0.36 (0.03- 4.12) | 0.39 |

**Table 14: Distribution of the TNF-α^-308^G>A (rs1800629) genotype frequencies with risk estimate in the BC prognostic models against good prognostic luminal A model (ER^+ve^PR^+ve^Her2^-ve^) in the BC group**

| 1. **Poor prognosis luminal B model (ER^+ve^PR^+ve^Her2^+ve^)** | | | | | | | | |
| --- | --- | --- | --- | --- | --- | --- | --- | --- |
| **Model** | **Genotype # (%)** | | | | **OR (95% CI)** | | **P** | |
| **Codominant** | **ER^+ve^PR^+ve^Her2^-ve^ 63 cases** | | **ER^+ve^PR^+ve^Her2^+ve^ 52 cases** | |  | |  | |
| **GG** | 12 (19) | | 8 (15.4) | | 1 | |  | |
| **GA** | 47 (74.6) | | 41 (78.8) | | 1.31 (0.48- 3.51) | | 0.39 | |
| **AA** | 4 (6.4) | | 3 (5.8) | | 1.12 (0.19- 6.43) | | 0.61 | |
| **Dominant** | AA + GA **vs GG** | | | | 1.29 (0.48- 3.45) | | 0.39 | |
| **Recessive** | **AA vs** GG + GA | | | | 1.1 (0.23- 5.18) | | 0.6 | |
| **Overdominant** | **GA vs** AA + GG | | | | 0.78 (0.33- 1.89) | | 0.37 | |
| 1. **Triple^–ve^ (very poor prognostic model)** | | | | | | | | |
| **Model** | | **Genotype # (%)** | | | | **OR (95% CI)** | | **P** |
| **Codominant** | | **ER^+ve^PR^+ve^Her2^-ve^ 63 cases** | | **ER^-ve^PR^-ve^Her2^-ve^ 11 cases** | |  | |  |
| **GG** | | 12 (19) | | 0 (0) | | 1 | |  |
| **GA** | | 47 (74.6) | | 10 (91) | | 1.21 (1.07- 1.36) | | 0.12 |
| **AA** | | 4 (6.4) | | 1 (9) | | 1.25 (0.8- 1.94) | | 0.29 |
| **Dominant** | | AA + GA **vs GG** | | | | 1.21 (1.08- 1.36) | | 0.12 |
| **Recessive** | | **AA vs** GG + GA | | | | 0.68 (0.07-6.7) | | 0.56 |
| **Overdominant** | | **GA vs** AA + GG | | | | 0.3 (0.03- 2.47) | | 0.22 |
| 1. **Her2-enriched poor model (ER^-ve^PR^-ve^Her2^+ve^)** | | | | | | | | |
| **Model** | | **Genotype # (%)** | | | | **OR (95% CI)** | | **P** |
| **Codominant** | | **ER^+ve^PR^+ve^Her2^-ve^ 63 cases** | | **ER^-ve^PR^-ve^Her2^+ve^ 14cases** | |  | |  |
| **GG** | | 12 (19) | | 2 (14.3) | | 1 | |  |
| **GA** | | 47 (74.6) | | 11 (78.6) | | 1.4 (0. 37- 7.2) | | 0.51 |
| **AA** | | 4 (6.4) | | 1 (7.1) | | 1.5 (0.11- 21.31) | | 0.62 |
| **Dominant** | | AA + GA **vs GG** | | | | 1.41 (0.27- 7.16) | | 0.5 |
| **Recessive** | | **AA vs** GG + GA | | | | 0.88 (0.09- 8.55) | | 0.64 |
| **Overdominant** | | **GA vs** AA + GG | | | | 0.8 (0.19- 3.24) | | 0.52 |
